# Identification of Learning Mechanisms in a Wild Meerkat Population

**DOI:** 10.1371/journal.pone.0042044

**Published:** 2012-08-08

**Authors:** Will Hoppitt, Jamie Samson, Kevin N. Laland, Alex Thornton

**Affiliations:** 1 Centre for Social Learning and Cognitive Evolution, School of Biology, University of St. Andrews, St. Andrews, Fife, United Kingdom; 2 Kalahari Meerkat Project, Kuruman River Reserve, Van Zylsrus, Northern Cape, South Africa; 3 Department of Zoology, University of Cambridge, Cambridge, United Kingdom; 4 Department of Experimental Psychology, University of Cambridge, Cambridge, United Kingdom; Université Paris 13, France

## Abstract

Vigorous debates as to the evolutionary origins of culture remain unresolved due to an absence of methods for identifying learning mechanisms in natural populations. While laboratory experiments on captive animals have revealed evidence for a number of mechanisms, these may not necessarily reflect the processes typically operating in nature. We developed a novel method that allows social and asocial learning mechanisms to be determined in animal groups from the patterns of interaction with, and solving of, a task. We deployed it to analyse learning in groups of wild meerkats (*Suricata suricatta*) presented with a novel foraging apparatus. We identify nine separate learning processes underlying the meerkats’ foraging behaviour, in each case precisely quantifying their strength and duration, including local enhancement, emulation, and a hitherto unrecognized form of social learning, which we term ‘observational perseverance’. Our analysis suggests a key factor underlying the stability of behavioural traditions is a high ratio of specific to generalized social learning effects. The approach has widespread potential as an ecologically valid tool to investigate learning mechanisms in natural groups of animals, including humans.

## Introduction

It is widely agreed that scientific endeavours to understand the evolutionary roots of human culture require knowledge of the extent to which the social transmission of information in human and non-human societies relies on homologous mechanisms [1], [2], [3]. Laboratory experiments can pinpoint the operation of specific mechanisms in captive animals, but cannot generate evidence that the same mechanisms operate in natural social groups, subject to all the stressors of life in the wild. Conversely, observations of natural behaviour alone cannot discriminate between alternative social (or asocial) learning mechanisms. Here we present a novel analytical tool that allows investigation of learning mechanisms in natural groups of animals (including humans) and apply it to a new dataset from groups of wild meerkats. Our methodology allows us to determine for the first time the social and asocial learning mechanisms operating in the wild, but the methods could also be applied to captive groups.

Traditional social learning experiments involve presenting a set of subjects, or “observers”, with the opportunity to observe one or more “demonstrator” animals that have been trained to perform target behaviour, usually the solution to a foraging task. The subjects’ performance is then assessed in a subsequent test phase, in which they are given access to the task, to ascertain whether acquisition of the behaviour has been improved as a result of the observational experience, compared to control subjects. This traditional social learning experiment design (henceforth ‘traditional approach’) has been modified in various ways to isolate different social learning mechanisms, taking advantage of the fact that the experimenter has a high degree of control over what social cues are available to the observers [4].

The traditional approach has been fruitful in establishing that certain species have a capacity for specific types of social learning [4]. However, the high level of experimental control comes at the cost of decreased ecological validity: the traditional approach does not allow the level of social interaction that would occur in freely interacting groups of animals. Consequently, the traditional approach can tell us little about the relative importance of different social learning mechanisms in such situations, or the role each one has in promoting or inhibiting the emergence and stability of traditions under natural conditions [5], [6]. For example, keas (*Nestor notabilis*) have been shown to use observational conditioning in captivity [7] but failed to do so in the wild [8]. Furthermore, whilst laboratory experiments on chimpanzees (*Pan troglodytes*) suggest an important role for imitation in tool use tasks [9], some field researchers [10] suggest local enhancement plays a dominant role in the acquisition of tool use in the wild. Similarly, social learning appears to be primarily restricted to the juvenile period in wild chimpanzees [10] but not restricted in this way for captive chimpanzees[9]. It is also conceivable that some species may not exhibit evidence of a capacity for a specific type of social learning unless presented with naturalistic social interactions. Finally many species are not amenable to study in the laboratory, and though approaches similar to the traditional approach are sometimes possible in the field [11], [12], this will not always be the case. This is a severe limitation if one’s goal is to obtain a picture of the taxonomic distribution of social learning mechanisms or understand the selection pressures driving their evolution.

Such concerns have recently led researchers to devise experiments and observational studies of the diffusion of innovations through groups of freely interacting animals [11], [13], [14]. These range from initiated diffusions (where groups are presented with a novel task) in captive and wild groups, to natural diffusions of spontaneously arising innovations. It has been noted that whilst ecological validity and the potential for understanding the factors affecting culture increases with increased naturalism, the potential for understanding social learning processes decreases [15]. Further experimental control is possible in initiated diffusions by “seeding” groups with demonstrators trained to solve the task using one of two or more different options: the researcher can then test whether the groups tend to adopt the same option as their demonstrator. However, in all diffusion experiments, the experimenter has, at best, very limited control over the social cues received by each individual, so information on these must be gathered as observational data [16]. Such data, collected on a fine temporal scale, is likely to contain statistical patterns indicative of different social and asocial learning mechanisms, but the analytical tools required to extract these patterns have, to date, been lacking.

Here we present a conceptual framework for the analysis of detailed observational data from seeded or unseeded diffusion studies (or indeed other social learning experiments) and present methods for detecting the presence of different mechanisms and quantifying their effects. We deploy a novel statistical approach. We call this a ‘stochastic mechanism-fitting model’ (henceforth ‘SMFM’) since it formulates hypothetical mechanisms as stochastic models, allowing us to assess the evidence for their presence and estimate the size and duration of their effects.

We applied the SMFM to data from a specially-designed initiated social diffusion experiment on wild meerkats. Meerkats are cooperatively breeding mongooses that have been the subjects of extensive studies of social learning under natural conditions [17]. However, the mechanisms by which information spreads through meerkat groups (or indeed social groups in any species) are unknown.

**Figure 1:**
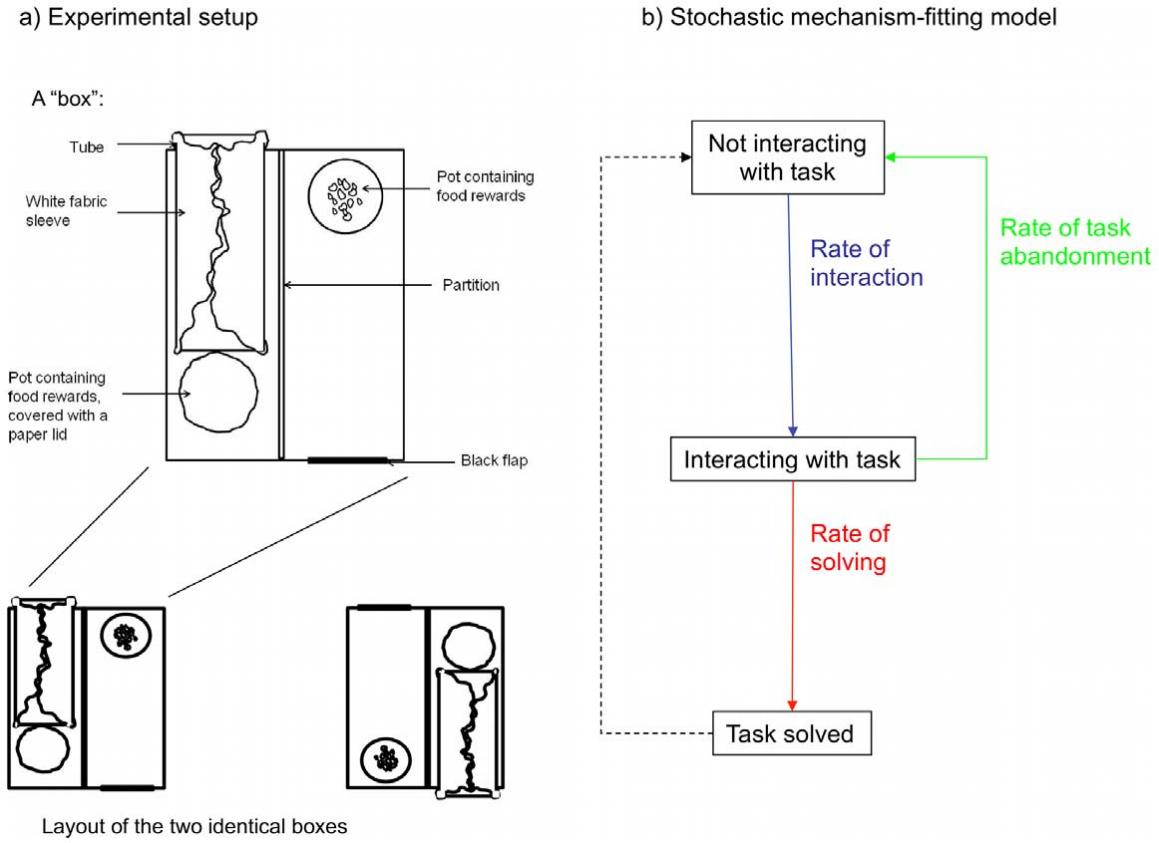
Experimental setup and model structure. A) A “Box”. The “Flap” technique involved going through a black cat flap to obtain food from a pot; the “Tube” technique involved pushing through a fabric sleeve on the tube and breaking a paper lid to obtain food; B) experimental layout of the two identical Boxes; C) diagrammatic representation of the stochastic mechanism-fitting model (SMFM) showing the three rates of transition that were modelled. In reality ‘rate of interaction’ involved modelling four ‘competing’ transition rates, to each of the four options available: left Flap, right Flap, left Tube and right Tube. We recorded an individual as solving the task when it gained access to food inside the box, and as abandoning the task when it terminated a bout of interaction without gaining access to food inside the box.

Demonstrator animals (subordinate adult male meerkats) were trained, out of sight of others, to obtain food from an experimental apparatus (hereafter a ‘Box’) using one of two ‘option-types’ (henceforth ‘Flap’ and ‘Tube’) positioned on opposite sides of a clear plastic box (Fig. 1A and B). The demonstrators then reliably performed their trained behaviour in front of a group of conspecifics over eight sessions, during which two identical Boxes were positioned 30cm apart, facing opposite directions (Fig. 1A), giving four possible ‘options’ for solving the task. Three meerkat groups were exposed to Flap-solver demonstrators, three to Tube-solvers, and a further three had no demonstrators (controls). We recorded the duration of all bouts of observation or interaction with the Boxes, noting the identity of the individuals involved, whether an individual observed another interacting with a Flap or Tube, whether it witnessed successful entry into a Box, whether food was obtained, and other relevant variables (see Materials and Methods for details). The two-Box design allowed us to distinguish between local enhancement effects (attraction to a particular location [18]) and stimulus enhancement (attraction to a particular stimulus type, such as black flaps or white tubes [19]), while other aspects of the method allow alternative learning mechanisms to be isolated (see below).

Historically, researchers have assumed that imitation and teaching may be necessary for stable cultural traditions [2], [20], [21], a view conflicting with recent empirical and theoretical work suggesting that stimulus and local enhancement can result in the formation of traditions [22], [23], [24], [25], [26]. Here we utilise a method that can be used to study these and other learning mechanisms in a natural context, and allow us to investigate, empirically, the relationship between learning mechanisms and the emergence of behavioural traditions.

We fitted stochastic models (see Methods and Materials) to the data, modelling individuals’ rates of transition between states of not interacting and interacting with each specified Box and Option (Fig. 1B). We modelled the rate at which an individual, *i*, initiated a bout of interaction with each Flap and Tube as a function of (*i*) individual differences in rate, (*ii*) *i*’s past successes using Flap and/or Tube (asocial learning), (*iii*) the observed number of entries by others to the Box using each option (direct social learning), and (*iv*) the latency since *i* observed another individual interacting with each option (transient social effects). We then used a stochastic model of the rate of interaction with the task in continuous time, in which the rate of interaction with each option was specified at a given time. Learning effects were modelled using an approximation to the Rescorla-Wagner learning rule, where association of an option-type with food increased to a maximum strength with repeated rewards. We derived a likelihood function and used Markov Chain Monte Carlo (MCMC) to generate posterior samples for the parameters in the model. We summarise the posterior sample using the median and 95% highest posterior density intervals (denoted as “95% HPD”), giving the range of probable parameter values. Where relevant we also provide posterior probabilities for statements regarding inequalities of parameters: for example 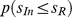 = 0.019 means that, conditional on the model, there is only a 1.9% probability that 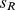 is less than or equal to 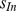. To explore factors affecting the rates of task solving and task abandonment we used Cox models, which have the advantage that they make no specific assumptions about the shape of latency distributions underlying the model [27]. We used a model averaging procedure to estimate effects, based on Akaike’s Information Criterion (AIC), and present back-transformed 95% unconditional confidence intervals (denoted “95% UCI”) [28]. Full details of the models and model selection procedure are given in the Text S1.

## Results

**Figure 2:**
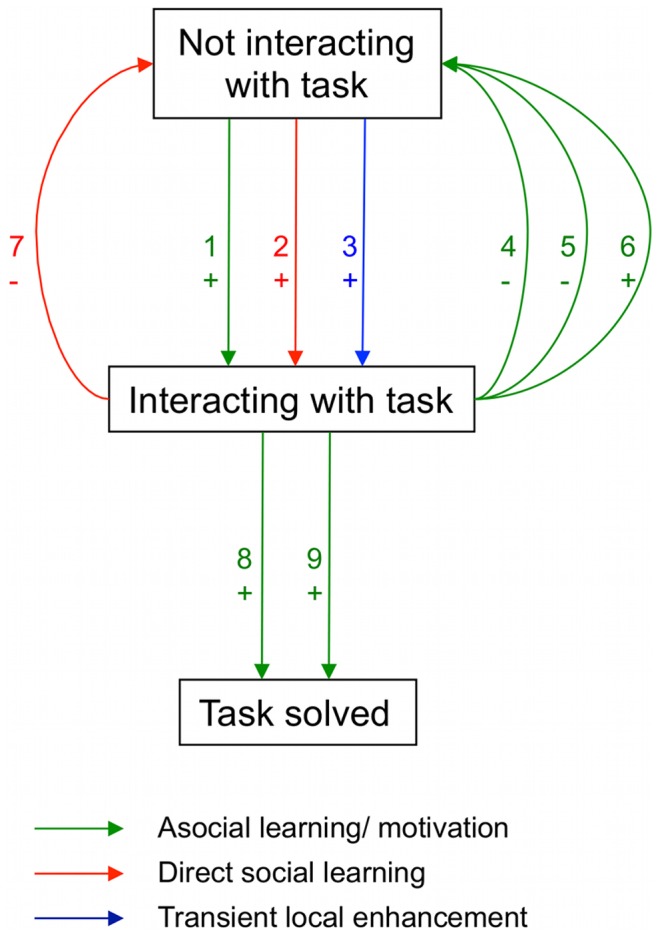
Diagrammatic representation of all effects found. Each effect is described and interpreted in Table 1. The positioning of the arrow for each effect represents the transition rate affected. Green arrows mean a rate of transition was found to be a function of an individual’s previous manipulations of the task, interpreted as asocial learning or changes in motivation. Red arrows mean a rate of transition was found to be a function of the number of previous observations, interpreted as direct social learning. The blue arrow indicates the rate of interaction was found to be a function of the time since last observation at each option, interpreted as a transient local enhancement effect. + or - indicates whether the transition rate was positively or negatively associated with the variable in question.

Excluding the six trained demonstrators, 77/170 meerkats manipulated the task with a total of 513 manipulations (mean = 6.7 per manipulator), 36 individuals were successful in obtaining food (i.e. were “solvers”) with a total of 271 successful manipulations (mean = 7.5 per solver). The models identified nine separate processes underlying the successful foraging behaviour of the meerkat groups, including three separate social learning processes and a further six asocial learning processes (Fig. 2, Table 1). In general, social factors played critical roles in drawing meerkats to interact with the apparatus, and keeping them at the task, while asocial learning processes dominated task acquisition.

**Table 1:** Summary of effects found on meerkats’ task solving behaviour, and our interpretation.

Label inFig. 2	Description of effect	Interpretation
1	Rate of interaction positively associated with number ofprevious successful interactions. Option-type specific.	Asocial learning. Interaction with an option-type is reinforced by successful interactions – a straightforward case of operant conditioning.
2	Rate of interaction positively associated with number ofprevious observations of conspecifics gaining entry to the box.Only weak evidence the effect is option-type specific.See Fig. S2.	Direct social learning. Not consistent with learning an association of the Box with food (‘observational conditioning’, sensu Heyes 1994), since seeing another individual feeding in box was not sufficient for the effect to occur. Perhaps individuals learned it was possible to get into the box, a case of emulation.
3	Rate of interaction higher in the period immediately afterobservation of a conspecific manipulating the task. Some effect onall options, but much stronger on the specific option observedto be manipulated, for younger meerkats. See Fig. 2.	Local enhancement: Observation of others manipulating the task transiently draws the observers to that location. The effect was more spatially specific for younger meerkats. See Text S1 for further investigation of this causal interpretation.
4	Rate of abandonment lower for individuals whohad previously solved the task. Option-type specific.	Asocial learning: interaction with an option-type is reinforced by a first successful interaction.
5	Rate of abandonment negatively associated with the numberof previous unsuccessful attempts to manipulate the task.Option-type general.	Individuals with more previous failures are hungrier, so more highly motivated to succeed once they start manipulating the task.
6	After accounting for effect 4, rate of abandonment positivelyassociated with the number of previous successful attemptsto manipulate the task. Option-type general.	Individuals with fewer previous successes are hungrier, so more highly motivated to succeed once they start manipulating the task, whilst individuals that have successfully retrieved lots of food become satiated.
7	Rate of abandonment negatively associated with the numberof previous observations of conspecifics gaining entry tothe box and feeding. Option-type general.	Direct social learning: observation of others solving the task caused meerkats to persevere with the task for longer during bouts of interaction. We term this ‘observational perseverance’.
8	Rate of solving higher for individuals who had previouslysolved the task. Option-type specific.	Asocial learning: the actions required to solve the task using a specific option-type are reinforced. Another instance of operant conditioning.
9	Rate of solving positively associated with the number ofprevious unsuccessful attempts to manipulate the task.Option-type specific.	Asocial learning: reinforcement of actions leading closer to task solution and/or punishment of actions not leading closer to task solution. Another instance of operant conditioning.

See also Fig. 2.

Three factors were found to increase the rate of interaction with the box (Fig. 2). The first was operant conditioning (Process 1 in Fig. 2, Table 1). The observed rate of interaction with the box by a given individual was found to be positively associated with their number of previous successful interactions, in an option-type specific manner. The estimated effect of each successful manipulation for an average (median) subordinate meerkat was 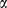 = 0.051; 95% HPD =  [0.040, 0.063], where 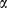 is the parameter that quantifies the learning rate in the Rescorla-Wagner model (see Eqn. 2). In contrast, dominant meerkats tended to be affected very little by operant conditioning (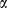 = 5.5E–12; 95% HPD =  [0, 9.9E–4]).

Second, we found that meerkats that observed a conspecific gain entry to the box (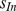 = 0.0035; 95% HPD = [0.0017, 0.0055]) themselves subsequently increased their rate of interaction with the box (Process 2 in Fig. 2, Table 1). Here and below, *s* terms can be viewed as social equivalents to 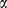. This observational effect was stronger than merely observing an individual feeding inside the box (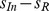 = 0.0028; 95% HPD =  [−5.1E-5, 0.0058]; 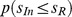 = 0.019), and elevated relative to individuals who did not observe the interaction at all (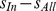 = 0.0028; 95% HPD =  [2.6E–5, 0.0054]; 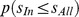
<0.001; see Fig. S2). However, we found no evidence that the effect was stronger for individuals who observed a conspecific both gaining entry to a box and receiving a reward (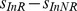 =  −5.2E–4; 95% HPD =  [−0.0050, 0.0045]; 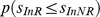 = 0.583), implying that observing a conspecific gain entry to the box was necessary and sufficient for direct social learning to occur. This effect generalised between option-types as observations of individuals gaining entry via the flap increased rates of interaction with the tube, and vice versa (see Fig. S1). However, there was weak evidence that the effect was stronger on the same option-type (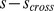 = 0.0022; 95% HPD =  [−1.1E–4, 0.0045]; 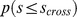 = 0.027; see Fig. S1). These observations rule out an interpretation of this form of observational learning in terms of local or stimulus enhancement, observational conditioning, imitation or response facilitation, and appear to be most consistent with the process of ‘emulation’ [29]. Broadly defined, emulation occurs when after observing a demonstrator interacting with objects in its environment an observer becomes more likely to perform any actions that bring about a similar effect on those objects [4]. Here, the meerkats appear to have learned through observation that it was possible to get into the box, and observation of others getting into the box makes them more likely to try to do so themselves.

**Figure 3:**
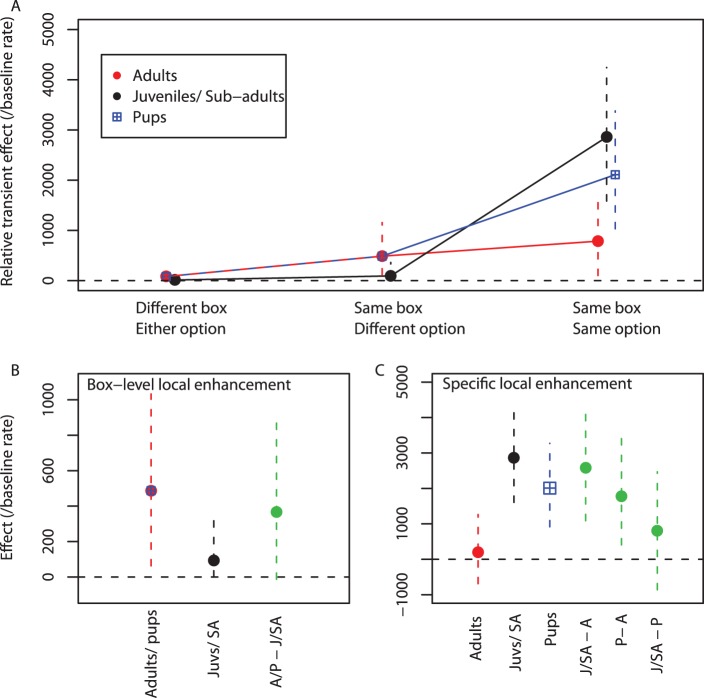
Estimated transient effects. A) Estimated size of the transient increase in rate of interaction at each option immediately following observation, for different age classes of meerkats (taken from the final model). These effects are decomposed into B) box-level local enhancement, influencing rate of interaction with both options at the manipulated box; and C) specific local enhancement, further influencing rate of interaction with the manipulated option. Estimates are the median of the posterior distribution, scaled relative to the estimated median baseline rate of interaction with the flap option. Error bars give the 95% highest posterior density (HPD) interval. Green points and error bars give the estimates of the difference in effect size between different age classes, where A = adult; J/SA =  juveniles and sub-adults; P = pups.

Third, we found that individuals were more likely to interact with all options on either Box immediately after observing a conspecific interacting with any one of them (see Fig. 3. Process 3 in Fig. 2, Table 1). We had allowed for the fact that observing others might transiently increase an observer’s rate of interaction with the box, which could indirectly result in social learning by influencing its asocial learning experience– for instance, through ‘stimulus enhancement’ or ‘local enhancement’ [4]. This was detected by including a component that was a function of the time since an individual had observed another individual interacting with each other option, assuming such effects decay exponentially in time (see Fig. S2). There was strong evidence that the effect was larger for the specific option and Box observed, indicating it was highly spatially-specific, and more pronounced in non-adults than in adults (see Fig. 3 and Table S1). This specific effect did not generalize to the same option-type on the other Box, ruling out stimulus enhancement, and strongly indicating an interpretation in terms of ‘local enhancement’. Local enhancement occurs when, after or during a demonstrator’s presence, or interaction with objects at a particular location, an observer is more likely to visit or interact with objects at that location [4], [18].

Our model also enables us to estimate the duration of the local enhancement effect. For an exponential model, this is intuitively captured by the half-life (time taken for the effect to halve in magnitude), which we estimated to be 20s (95% HPD = [12], [29]). To our knowledge this is the first precise estimate of the duration of local enhancement, although experimental studies have determined that local enhancement effects persisted for greater than a fixed interval (e.g. [30]). We suggest that determining whether a social effect persists for greater than a fixed interval is not a particularly good way of quantifying its duration. Whether or not we can detect an increase relative to baseline is as much a function of sample size as the nature of the process. If we had a very large sample size we might conclude that local enhancement lasts for a very long time: however, the estimated effect at this point would likely be so small as to be unimportant. It makes more sense to ask how fast the effect fades- the precision of this estimate is then a function of sample size. In addition, we can investigate the conditions under which local enhancement occurred by fitting alternative models and comparing deviance information criterion (DIC) values. We tested for transient effects conditional on observation of a conspecific gaining entry to the box (ΔDIC =  +168.2), and observation of a conspecific obtaining a reward (ΔDIC =  +137.8). We also fitted a model in which the transient effects operated on all individuals present at an experimental session, regardless of whether they were recorded as an observer (ΔDIC =  +399.2). All alternative models provide a worse fit to the data, suggesting that observation of a conspecific interacting with an option was a necessary and sufficient condition for the transient social effects to occur.

We estimated that meerkats that had previously solved the task subsequently solved it at a 50% higher rate (Process 8 in Fig. 2, Table 1: x1.51; 95% UCI =  [1.00, 2.01]) and abandoned the task at a third of the rate (Process 4 in Fig. 2, Table 1: x0.34; 95% UCI =  [0.23, 0.49]) during future manipulations of the same option-type. Counter-intuitively, the rate of task abandonment increased with the number of further previous successes at either option-type (Process 6 in Fig. 2, Table 1: x1.09 each successful manipulation; 95% UCI =  [1.04, 1.14]) perhaps due to decreased motivation, with the meerkats having become satiated. In addition, the number of previous unsuccessful interactions was negatively associated with the rate of abandonment (Process 5 in Fig. 2, Table 1: x0.84 each unsuccessful manipulation; 95% UCI =  [0.74, 0.96]; option-type general) and positively associated with the rate of solving (Process 9 in Fig. 2, Table 1: x1.12 each successful manipulation; 95% UCI =  [1.01, 1.25]; option-type specific) suggesting individuals might acquire useful information from unsuccessful manipulations. This latter finding is consistent with findings that the ‘error’ can be crucially important to effective trial-and-error learning.

While there was little evidence that observation of others directly affected the rate of solving the box task (see Table S2), an individual’s rate of task abandonment declined with the number of successes it had observed (Process 7 in Fig. 2, Table 1: x0.84 each observation; 95% UCI =  [0.76, 0.94]), suggesting that observing the success of others decreased the rate at which individuals gave up on the task. There was strong evidence that this effect required observation of a conspecific both gaining entry to the box and obtaining a food reward and that the effect was not option-type specific (see Table S3). To our knowledge, this effect of social learning has not previously been detected in any previous human or animal experiment. As the effect of observing others’ successes appears primarily to encourage individuals to persist with the task, in the absence of a recognized label we have termed this process ‘observational perseverance’.

**Figure 4:**
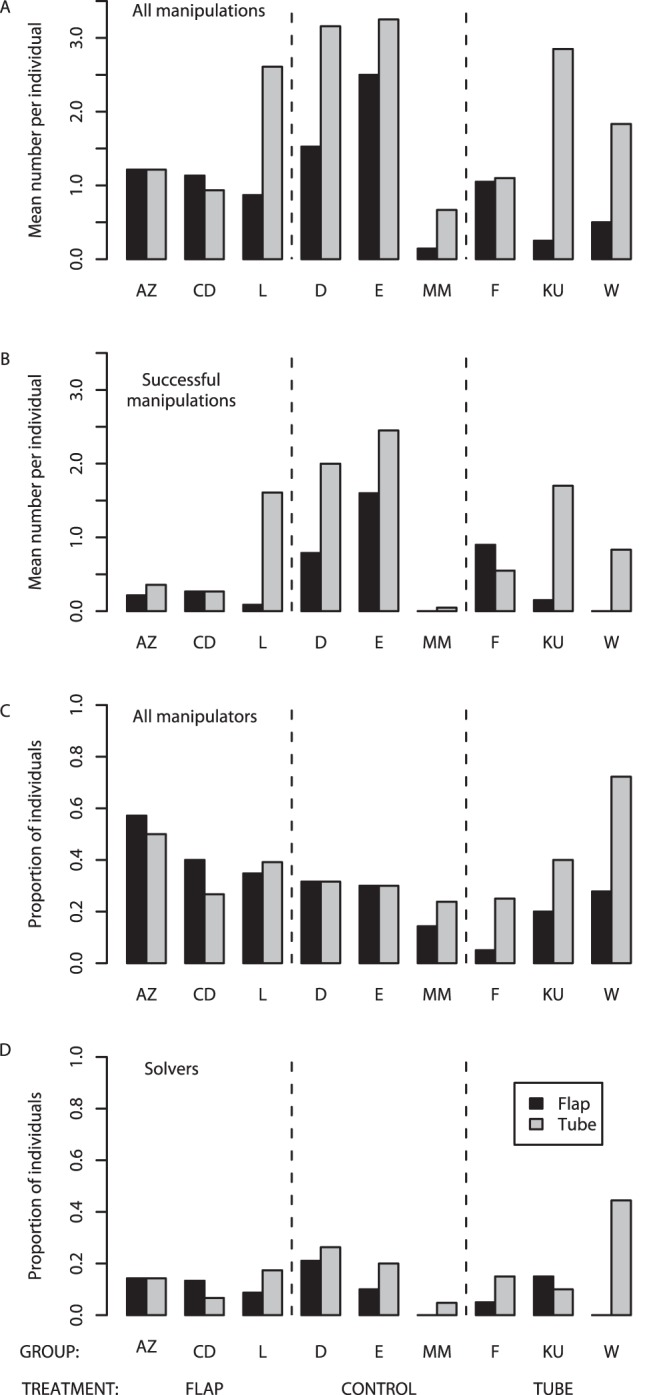
Group differences in manipulations of the flap and tube. A) The number of manipulations of the flap and tube; B) the number of successful manipulations of the flap and tube; C) the proportion of individuals that manipulated the flap and tube; and D) the proportion of individuals solving the task using the flap and tube. Trained demonstrators are not included in all cases. Letter codes refer to different groups.

**Figure 5:**
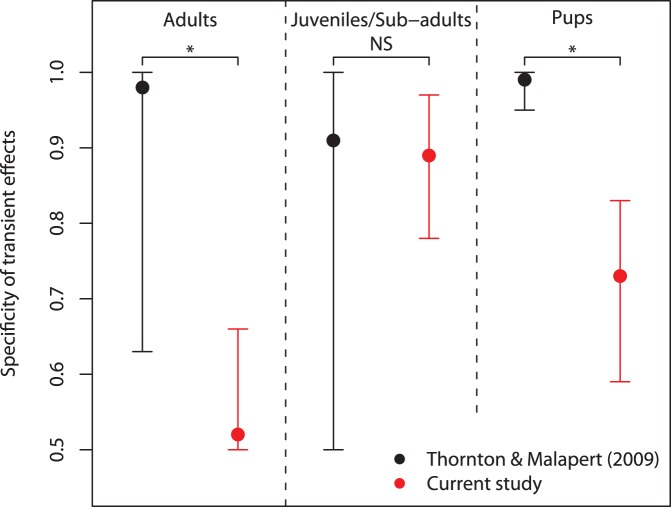
Specificity of the transient social effect for different age classes for the current study and the previous experiment by Thornton and Malapert [**21**]. Specificity quantifies the probability a naive observer will use the same option-type it has observed, given that it manipulates one of them immediately after observation. The mean of the posterior sample is shown in each case, with the 95% central interval. * Indicates that the 95% central interval for the difference between the two studies did not include zero, whereas NS signifies that it did.

Despite strong evidence of social learning processes affecting the learning of wild meerkats, the demonstrators’ techniques did not spread to form strong group-level traditions (see Fig. 4, modified Option Bias test [31]; *P* = 0.080; see Text S1 for details). We suggest that in this study the ratio of specific to generalized local enhancement effects was too low to promote the maintenance of the demonstrated option. Had the dominant social learning effects been more strongly option specific, rather that generalizing to other options, then traditions may have been detected. As a test of this hypothesis, we applied the SMFM method to experimental data reporting stronger evidence of group-level traditions in meerkat groups [5] and, as predicted, found the estimated transient effects were more specific to the observed option-type (Fig. 5; see Text S1).

## Discussion

Groups of wild meerkats were found to solve a novel foraging task through an interwoven complex of nine separate processes, including three types of operant conditioning and three separate forms of social learning. With respect to the latter, we found that observation of others interacting with and solving the task made meerkats more likely to succeed in a given bout of interaction with the task. This is unlikely to be a result of imitation (copying a motor pattern), since observing successful manipulations did not raise observers’ solving rate disproportionately when using the same option type, nor did it improve the rate at which they solved the task during a bout of interaction. Rather, observation of others’ successes caused individuals to interact with the task at higher rates and to persist for longer once they had begun a bout of interaction, with attention being transiently drawn to specific variant solutions. The dominant social influence was a specific local enhancement effect, which attracted individuals to the exact option and Box with which they observed another individual interacting, but emulation and observational perseverance also played a role. While it is known that influences on perseverance may in turn affect learning (e.g. [32]) the role of social observation in mediating perseverance and hence the acquisition of new skills has not previously been described. Moreover, although stimulus and local enhancement are commonly thought of as cognitively unsophisticated, an understanding of simple mechanisms is central to our understanding of cognitive evolution [33]. Laboratory studies commonly infer local and stimulus enhancement when evidence for imitation is lacking, but seldom discriminate between them, examine the magnitude or duration of these effects, specify the conditions under which they occur, or describe how they interact with other asocial and social learning processes. Nor, unlike the SMFM approach, do established social learning methodologies typically identify multiple learning processes underlying a particular bout of behaviour. Accordingly, the insights gained from this study go significantly beyond conventional studies of social learning, or the detection of local enhancement in the laboratory.

Fig. 2 and Table 1 provide a summary of the effects found, and our causal interpretation. Whilst not all effects detected map easily onto existing terminology for social learning mechanisms, this terminology is based primarily on the study of animals in artificial (i.e. laboratory) contexts, and existing classification schemes are widely thought to be incomplete, with overlapping and non-hierarchical categories, and with evidence for several processes contentious (e.g. [4], [34]). The processes isolated here have the advantage that they are known to be deployed in a natural context by wild animals. We are also able to infer the conditions for each effect to occur, and the consequences this has for an individual’s future behaviour. As such, our SMFM approach might yield important insights into the limitations of primarily laboratory-based terminology (e.g. [4], [35]) for describing those learning mechanisms actually deployed by animals in a natural social and ecological context. Perhaps more importantly, the SMFM framework allows for the fact that information transmission in animal groups might reflect a composition of multiple mechanisms, and provides a means for disentangling and quantifying the mechanisms’ individual effects in both laboratory and field studies.

Moreover, our SMFM analysis detects strong evidence for social learning processes affecting the learning of wild meerkats, despite the fact that demonstrators’ techniques did not spread to form group-level traditions. This has two important implications. First, researchers deploying conventional tools reliant on finding between-group differences in behaviour to infer social learning are probably failing to detect many instances of social learning in natural animal populations. This is consistent with recent empirical findings suggesting that, contrary to common assumption [31], social learning need not lead to within-group homogeneity [5], [12], [36]. The SMFM approach has the advantage that it detects social influences on learning regardless of whether they result in population differences in behaviour. Second, by linking mechanisms to social behaviour, the SMFM approach is able to explain why, in this instance, traditions did not form. We suggest that in this study the ratio of specific to generalized local enhancement effects was too low to promote the maintenance of the demonstrated option. Had the dominant social learning effects been more strongly option-type specific, rather that generalizing to other option-types, then traditions may have been detected.

While it is possible that the operation of mechanisms such as imitation may allow greater fidelity in the transmission of information [2], our analysis suggests that other factors are potentially important, consistent with recent experiments on humans, which suggest that faithful transmission and cumulative cultural change may occur in the absence of imitation [37]. Our analysis implies that the persistence of traditions is more dependent on whether the social learning processes deployed are highly option specific, thereby failing to generalize to other solutions to the task in hand, rather than on the mechanism through which social learning occurs. Researchers have frequently assumed that the occurrence, persistence and complexity of behavioural traditions in different species reflect alternative underlying learning mechanisms [1], [2], yet hitherto it has not been possible to test this. It is also widely assumed that human cultural traditions are maintained through imitation and teaching [2], [20], [21], and that the greater stability of human traditions compared to those of other animals reflects a reliance on different learning mechanisms, but these assumptions are also virtually never tested. Our findings raise the possibility that human cumulative culture may require mechanisms that promote specificity in the solution adopted, such as conformity and punishment of violators of social norms [38], rather than, or as well as, high fidelity of information transmission. We suggest that the analytical tools presented here provide the means to meet these challenges and thus to develop a fuller understanding of the relationship between human and animal culture.

We note that application of SMFM to different task designs would allow researchers to distinguish between further mechanisms. For example, imitation and emulation could be distinguished if two options involved different motor patterns, but resulted in the same movements of the task (a ‘two-action test’ [39]). The SMFM would detect whether social influences are option-specific (indicating imitation), as well as providing additional information about which transitions are influenced, the time course of the effect, and the conditions under which it arises. The approach could also be generalized to apply to natural, rather than experimentally induced, traditions in animals, with particular utility where multiple options are observed (e.g. alternative ant-dipping methods by chimpanzees, *Pan troglodytes* [40], or variant tools used by New Caledonian crows, *Corvus moneduloides* [41].

Whilst existing approaches allow inferences to be made about the way in which individuals use social information to solve a task, to our knowledge none do so at a sufficient level of detail to allow specific psychological mechanisms to be identified. For instance, Kendal et al [42] provide a method for quantifying the extent to which social learning influences the rate at which individuals approach and subsequently solve novel tasks. However as this method is applied at the level of the group, it cannot take into account the dynamic nature of skill acquisition, whereby an individual’s competence changes over time in relation to its specific previous experience. In contrast, McElreath et al [43] model individuals’ choices between alternative options as a function of their previous observations of others’ choices and the reward obtained, thus allowing inference about the social learning strategies being employed. However, this approach is only able to detect learning mechanisms that influence option choice, and not those that influence rate of interaction, success or task abandonment. Our approach also differs from the recent use of multistate Markov chain models to model animal behaviour [44] since the rates of transition between states are a function of each individual’s past experience. Nonetheless, all of these studies share with SMFM the strategy of formulating hypotheses about behavioural mechanisms as stochastic models, which can be fitted to, and evaluated by time-structured data. We feel this under-used approach is likely to prove particularly fruitful in the study of animal behaviour [45].

A stochastic modelling approach could allow researchers to study mechanisms of behaviour in the wild, including in species that are not amenable to experimental manipulation. This would allow comparisons to be made across a wide range of species, not just convenient laboratory models or species for which field experiments are feasible. This approach could be of particular utility for the study of all aspects of social behaviour (e.g. communication, grouping, social networks, agonistic and affiliative encounters), where it can be difficult to manipulate the social cues received by an individual experimentally, in either the lab or the field. There is also considerable potential for applying similar techniques to analyze aspects of human behaviour within the social sciences. Thus the approach has widespread potential as an ecologically valid analytical tool with which to investigate learning mechanisms in natural groups of animals, including humans.

## Materials and Methods

### Ethics Statement

All data collection was carried out following Association for the Study of Animal Behaviour guidelines, with ethics approval from the Universities of Cambridge and Pretoria, under Northern Cape Conservation Authority Permit ODB 2575/2009.

### Study Site and Meerkat Population

Experiments were conducted between January and May 2009 on nine groups of 12–24 free-living meerkats (176 total) in the Kuruman River Reserve in northern South Africa. All individuals were habituated to close observation (<1 m) and could be recognised through unique marks of hair dye on their fur. Groups were located by radio-tracking one collared individual in each group. Whilst the meerkat population is habituated to human observers, it is entirely wild and thus subject to intense predation and food restriction [46], [47], and unlike captive groups, the meerkats exhibit natural social dynamics including dispersal, eviction, inter-group encounters and infanticide [48]. Crumbs of egg are used to attract meerkats onto scales for weighing as part of a long-term study, but these crumbs are <1 g (typically less than 0.15% of body weight). Rates of predator attack may be lower while researchers are present, but observers are not present continuously and survival rates are still lower than in related species [46].

### Experimental Apparatus

All experiments used identical “Box” apparatus (Fig. 1 a, Fig. S3). A Box consisted of a rectangular plastic box 37.5 cm long, 26.5 cm wide and 15 cm high. One face of the box had a black cat flap, hinged at the top, while the opposite face had a plastic tube which led into the box and protruded 2 cm from the face diametrically opposite to the flap. The tube was lined with a baggy, white fabric sleeve that blocked visual access to the inside of the box. Meerkats could either go through the flap to obtain food (crumbs of hard-boiled egg and pieces of freshly-killed scorpion) from a clear plastic pot (“Flap technique”) or push through the sleeve into the tube and rip apart a kitchen paper lid to access food from another pot (“Tube technique”). Boxes were made of clear plastic with perforations to allow individuals to see and smell the contents.

### Training Demonstrators

One demonstrator in three groups was trained on the Flap technique, and one demonstrator in another three groups was trained on the Tube technique. A further three control groups had no demonstrators. All demonstrators were subordinate adult males. We ensured that only demonstrators were exposed to training by conducting training sessions when demonstrators were foraging out of sight of the rest of the group or when demonstrators were babysitting pups that were underground at the breeding burrow while the rest of the group was foraging elsewhere. Demonstrators typically required five days of training to reach proficiency in either technique (4–9 training trials per demonstrator). Once demonstrators were fully trained (successful completion on five subsequent presentations), we conducted one training trial with two identical boxes, facing opposite directions. In all cases, demonstrators successfully obtained food from both boxes using their trained technique.

#### Flap training

We began by propping open the flap and leaving a trail of food leading into the box. We then incrementally closed the flap so that the individual had to push against it to enter the flap. Training ended once individuals reliably approached the box, pushed through the flap to obtain food from the pot inside and subsequently exited the box.

#### Tube training

We trained demonstrators on the Tube technique by first enticing them to go through the tube (with no sleeve) and obtain food from the pot inside. We then attached the sleeve and made it increasingly baggy until the sleeve obscured the view into the tube and the demonstrator had to push through it to go through the tube. Once demonstrators were reliably going through the tube in this manner, we began affixing a kitchen paper lid to the pot containing rewards. Initially, the lid was loosely attached on one side, so that the individual could put its paw under the paper to scoop out food. As individual grew more competent at this technique, we began to attach the paper more securely on all sides so that paper had to be ripped to access the food. Training ceased once demonstrators reliably approached the box, pushed through the tube, broke the paper lid and consumed food.

#### Control groups

To ensure that individuals in control groups were not afraid of the Boxes, we conducted a session prior to the group phase where the two boxes were placed on the floor and could be seen by all group members. No meerkats displayed mobbing behaviour, produced alarm calls or showed any fearful response to the Boxes, and no individuals attempted to enter the boxes.

### Group Phase

Once demonstrators were trained, group sessions were conducted during the morning period when all group members were present at the sleeping burrow before setting out to forage. Meerkats do not eat during the night, so motivation to obtain food should be comparable for all individuals. Two identical boxes, 30cm apart and facing opposite directions were placed adjacent to the sleeping burrow, visible to and approximately equidistant from all group members. Sessions lasted 3–35 mins (mean  = 19 mins ±0.9), depending on how long the group spent at the burrow, with sessions ending once the first individual moved more than 20 m from the burrow. We conducted eight sessions at each group, with sessions spaced at least three days apart (mean days between sessions  = 11±0.5). For one group, MM, we conducted an additional ninth session so that the total duration of all sessions was comparable (within 20 mins) at all groups. In all experimental groups the trained animals successful demonstrated the target behaviour proficiently.

Sessions were videorecorded using a Panasonic NV-GS80 camcorder (Panasonic Corporation, Kadoma, Japan). From the videos, the duration of all bouts where an individual interacted with a box or observed another meerkat interacting with a box were later transcribed by AT and JS. Bouts of interaction and observation could be coded unambiguously, and independent coding of the first five group sessions by AT and JS showed interobserver reliability of >95%. An interaction bout refers to a discrete period spent interacting with the apparatus (scratching, pushing or otherwise manipulating it). Interaction bouts commenced when a meerkat made physical contact with the apparatus and ended when the animal moved away from the apparatus. During interaction bouts, we noted which part of which box the individual interacted with (flap, tube or other), whether it entered the box and whether it obtained food. We recorded an individual as solving the task when it gained access to food inside the box, and we refer to the bout of interaction leading to this as a successful interaction. Observation bouts were defined as occurring when a meerkat was within 1 m of, and had its head oriented towards, another individual that was interacting with the box. During observation bouts, we noted whether an individual observed another interacting with a flap or tube and whether it witnessed successful entry into the box and/or acquisition of food. Whenever an individual ripped a paper lid or consumed the majority of the food in a box, we waited for it to leave and then rapidly removed the box (<10 secs), affixed a pre-prepared replacement paper lid and replenished the food before placing the box back in its original position.

### Data Analysis

Full details of the model, model selection procedure and causal interpretation of the model can be found in Text S1. Here we give a brief overview of the stochastic mechanism-fitting model. In sum, we derived a likelihood function and used Markov Chain Monte Carlo (MCMC) to generate posterior samples for the parameters in the model, using WinBUGS 1.4 [49], which were analysed using the coda [50] package in the R statistical environment [51].

#### Stochastic model of interaction with the task

We modelled the rate at which individuals initiated bouts of interaction with option-type k (flap = 1, tube = 2), on box l (left = 1, right = 2) for individual i in group j at time t in session s as:



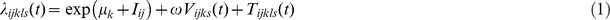
where 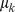 determines the rate of interaction for option-type k, 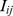 is a linear function of time-constant variables influencing i’s baseline rate of interaction with the task (age-class, sex, dominance and individual and group-level random effects), 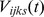 is i’s association of option-type k with reward, which is a function of past asocial and direct social learning (see below), 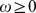
is a parameter determining the relative influence of learning, and 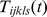 is a function describing transient social effects on i’s rate of interaction with option-type k, on box l at time t during session s (see below).

Learning in the model was based on the established Rescorla-Wagner learning rule [52], where a rewarded interaction with k by individual i in group j, increments its association with that option-type as follows:



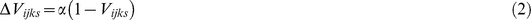
where 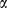 is a parameter controlling how quickly the maximum association is attained. This can be approximated, and extended to include the direct effects of observation as follows:



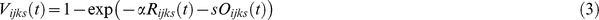
where 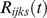 is the number of times i has been rewarded for interacting with k prior to time t in session s and all previous sessions.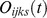 is the number of observations by i of interactions with k prior to time t in session s and in all previous sessions and s controls the strength the social learning in a manner analogous to 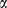. This means that inferences regarding *s* assess the evidence that observation of another individual solving the task exerts a permanent influence on the observer’s future rate of interaction with the flap and tube, as oppose to a transient effect (see below). We further generalised learning to investigate the conditions under which direct social learning occurred, by distinguishing different types of observation events, and allowing the rate of social learning to vary between them (e.g. 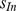 denotes the effect of observing a conspecific gain entry to the box).

We modelled transient social effects these effects by taking 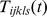 to be a function of the time since the times since another individual had last interacted with each option at each box within that session. We assumed that each of these effects would be strongest while a conspecific was interacting with the option in question, and fade away to baseline levels as time went on. For example, we modelled the effect of observation of a conspecific at the same option-type on the same box (SOSB) effect as follows:



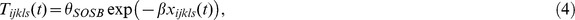
where 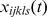 is the time since the last observation of a manipulation by individual i in group j, during session s of option-type k on box l, excluding manipulations by i, with 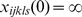, 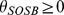
gives the strength of the SOSB effect, and 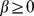 is the rate at which transient social effects die away, with 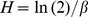 giving the half-life of the effects. We expanded the model to include transient effects operating across options and used the contrasts between these effects to distinguish local and stimulus enhancement. For example, stimulus enhancement would be inferred if observation increased interaction with the same option-type on a different box (SODB) more than the different option-type on the different box (DODB), i.e. 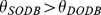
_._ We further expanded the model to include interactions of asocial learning, direct social learning, and transient effects with age-class, sex and dominance. Details of the final model are given in Table S1. In the results we give 95% highest posterior density (HPD) intervals for parameters and contrasts of interest, taken from the final model or from a model with unimportant effects added back in.

#### Modelling probability of successful manipulation

To model the probability that an individual would be successful (i.e. obtain food) in a given bout of manipulation with the task we used a GLMM with a binomial error structure and logit link function, with nested random effects for group and individual. We allowed for a difference in difficulty between flap and tube and tested for between-individual differences in the probability of success between males and females, pups, juveniles, sub-adults, subordinate adults and dominant adults. We also tested for how probability of success depended on an individual’s prior experience. As before, we assumed that potential influences could be *a*) an individual’s own history of manipulations, i.e. the cumulative number of successful interactions and number of unsuccessful interactions at the option being manipulated; *b*) direct social learning: a permanent effect resulting from observation, i.e. the cumulative number of observed successful manipulations at each option, and *c*) transient social influence, i.e. the time since another individual last interacted with the same option at the same box. This particular transient effect was chosen as the most likely to be in operation in light of its dominant effect on the rate of interaction.

Models were fitted using the lmer function in the lme4 package [53] of the R statistical environment [51], using the Laplace approximation. We fitted models including every combination of fixed effects, using R code that fitted each model and recorded the AIC (Akaike’s Information Criterion) in each case. This allowed us to judge the evidence for each behavioural mechanism based on its total Akaike weight, and provide model-averaged estimates for supported effects (see Text S1 for details) [28].

Changes in the probability of success in a bout could logically be the result of only two factors: *a*) changes in the rate at which individuals terminate a bout of interaction unsuccessfully, henceforth ‘task abandonment’; or *b*) changes in the rate at which individual terminate a bout successfully, henceforth ‘rate of solving’. To investigate how each variable operated, we fitted a separate model of each process, using a Cox Proportional Hazards survival analysis model [27]. For *a*), the time of ‘death’ is the time since initiating a bout at which an individual terminates a bout without gaining a reward. Those individuals who gain a reward are considered to be ‘censored’, equivalent to surviving the course of a survival analysis. Conversely, for *b*) the time of ‘death’ is the time since initiating a bout at which an individual terminates a bout by gaining a reward. In this case, those individuals who do not gain a reward are ‘censored’. The models were fitted using the coxme function in the coxme package [54] in the R statistical environment [51]. For each of a) and b) we used the same model averaging procedure as above, calculating AIC using the integrated likelihood. In the results we report 95% unconditional confidence intervals for parameters of interest, allowing for model selection uncertainty across all other variables.

## Supporting Information

Figure S1
**Mean and 95% central interval of the posterior distribution for the direct social learning effect of different classes of observation.** The size of each effect refers to the corresponding parameter in the learning rule used in the model (Eqn. 6, with constraints 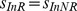 and 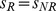). * indicates that the 95% central interval for the contrast between two effects did not include zero; NS indicates that the 95% central interval for the contrast between two effects did include zero. Note that only the effects for “Box entry observed” were retained in the final model.(EPS)

Figure S2 **Plot giving an unconstrained estimate of the shape of the transient function for the specific local enhancement effect.** This was computed by i) summing, across all individuals, the number of bout initiations within a given time period of observation of a conspecific at that same option; ii) summing, across individuals and options, the total time for which each individual was within a given time period of observation of a conspecific at each option: the ‘time available’, iii) estimating the rate for each time period by dividing the bouts initiated by the time available. The width of each interval was chosen such that it contained a minimum of 10,000s of time available, with the exception of the final interval, 1210+ s (5029s available time). The dashed line gives the rate before an individual had observed another individual interacting with a given option in that session (886 initiations, 5.4e+06 s available time), taken in the model as infinity.
(EPS)

Figure S3 **Photograph of the “boxes” used in the diffusion experiment, showing the tube (left) and flap (right) option types.**
(JPG)

Text S1 **Supplementary Information including a full specification of the SMFM, model fitting and selection procedure, causal interpretation of the results and further analyses run.**
(PDF)

Table S1 **Descriptive statistics of MCMC samples of the posterior distribution for parameters and contrasts in the final model.** 95% credible intervals are highest posterior density (HPD). Where relevant the posterior probability is given that the parameter or contrast ≤0. These are not given in cases where parameters were constrained to be >0 or where the hypothesis  = 0 is of no interest. Posterior probabilities are given to 3 d.p and in bold when <0.025. All other figures are given to 3 sig. figs.
(PDF)

Table S2 **Relative support for different models of the effect of direct social learning on rate of solving.**
(DOC)

Table S3 **Relative support for different models of the effect of direct social learning on rate of task abandonment.**
(DOC)
