# Supplementary figures and images for: Identification of Learning Mechanisms in a Wild Meerkat Population
Source: PLoS One. 2012 Aug 8;7(8):e42044. doi: 10.1371/journal.pone.0042044 (PMC3414518; doi:10.1371/journal.pone.0042044)

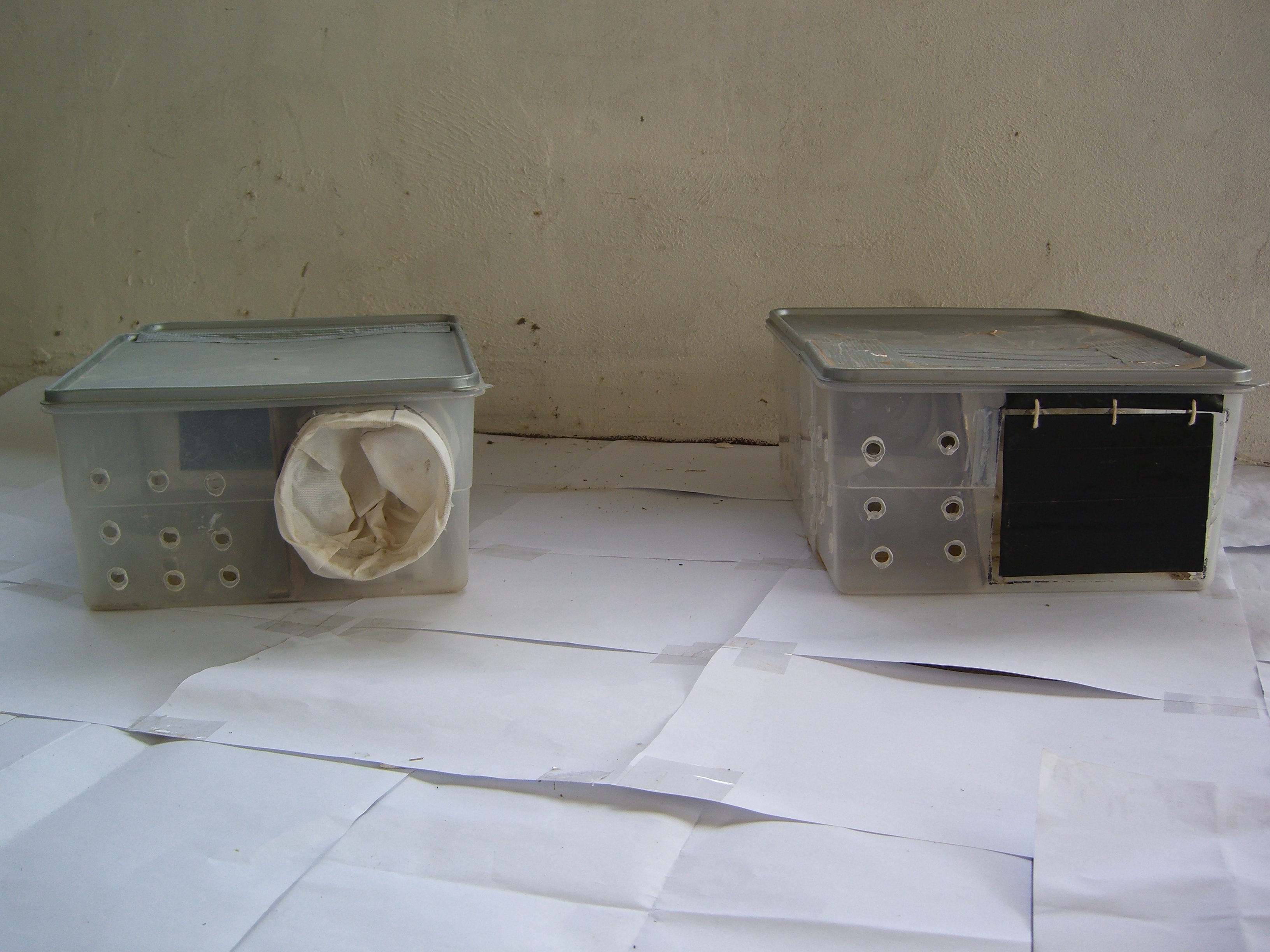

Supplement: Figure S3 — Photograph of the “boxes” used in the diffusion experiment, showing the tube (left) and flap (right) option types. (JPG) [file pone.0042044.s003.jpg]
