# Supplementary material for: Identification of Learning Mechanisms in a Wild Meerkat Population
Source: PLoS One. 2012 Aug 8;7(8):e42044. doi: 10.1371/journal.pone.0042044 (PMC3414518; doi:10.1371/journal.pone.0042044)
Supplement: Text S1 — Supplementary Information including a full specification of the SMFM, model fitting and selection procedure, causal interpretation of the results and further analyses run. (PDF) [file pone.0042044.s004.pdf]

SUPPLEMENTAL INFORMATION FOR

IDENTIFICATION OF LEARNING MECHANISMS IN A WILD MEERKAT POPULATION

Hoppitt, W, Samson, J., Laland, K.N and Thornton, A.

1. Stochastic model of interaction with the task

We modelled the rate of interaction of option-type k (flap=1, tube=2), on box l (left=1, right=2) for individual i in group j at time t in session s as:

$$\begin{aligned}
&\lambda_{ijkls}(t) = \left(\exp\left(\mu_k + I_{ij}\right) + \omega V_{ijks}(t) + T_{ijkls}(t)\right) p_{ijs} f_{ijs}(t) \\
&i = 1,2,...,N_j \\
&j = 1,2,...,G \\
&k = 1,2 \\
&l = 1,2 \\
&s = 1,2,...,S_j \\
&\omega \geq 0
\end{aligned} \quad , \qquad 1$$

where G=9 is the number of groups, N_j is the number of individuals in group j, S_j is the number of experimental sessions for group j, μ_k is the baseline rate of interaction for option k, I_{ij} is a linear function of variables influencing i's baseline rate of interaction with the task (see below), $V_{ijks}(t)$ is i's association of option k with reward, which is a function of past asocial and social learning (see below), ω is a parameter determining the relative influence of learning, $T_{ijkls}(t)$ is a function describing transient social effects on i's rate of interaction with option k, on box l at time t during session s (see below), p_{ijs} indicates whether i was present (1) or absent (0) for session s, and $f_{ijs}(t)$ whether i was potentially free to interact with the task (1) or whether it was already engaged in a bout of manipulation with a flap or tube (0)[1].

Individual-level variables

We considered a number of categorical variables: sex, age category: pup, juvenile, sub-adult and adult, with adults split further into subordinate and dominant individuals. The effects of these variables on individual i in j's, baseline rate of interaction were modelled as follows:

$$\begin{aligned}
&I_{ij} = \kappa_{male} male_{ij} + \kappa_{pup} pup_{ij} + \kappa_{subad} subad_{ij} + \kappa_{juv} juv_{ij} + \kappa_{dom} dom_{ij} + \eta_{ij} + \nu_j \\
&\eta_{ij} \sim N\left(0, \tau_{ind}\right) \\
&\nu_j \sim N\left(0, \tau_{group}\right)
\end{aligned} \quad , \qquad 2$$

[1] p_{ijs} and $f_{ijs}(t)$ are omitted from the overview of the model in the main text for clarity.

where $male_{ij}$, pup_{ij}, juv_{ij}, $subad_{ij}$ and dom_{ij} are indicator variables which take the value 1 if individual i in group j is male, a pup, a juvenile, a sub-adult or a dominant individual respectively, and 0 otherwise. κ terms are fitted parameters determining the effects of each of these variables. Thus rates for each category are determined relative to a subordinate adult female. η_{ij} is a random effect controlling for within individual correlation not accounted for by the other variables in I_{ij}, and ν_j is a random effect controlling for correlation between individuals in the same group.

Asocial Learning and Direct Social Learning

Learning in the model was based on the established Rescorla-Wagner learning rule, where a rewarded interaction with option k by individual i in group j, increments its association with that option as follows:

$$\Delta V_{ijks} = \alpha\left(1 - V_{ijks}\right), \quad \alpha \geq 0 \qquad 3$$

where α is a parameter controlling how quickly the maximum association is attained. This can be approximated as follows:

$$V_{ijks}(t) = 1 - \exp\left(-\alpha R_{ijks}(t)\right), \qquad 4$$

where $R_{ijks}(t)$ is the number of times i has been rewarded for interacting with option k prior to time t in session s and all previous sessions. This can be extended to include direct social learning as follows:

$$V_{ijks}(t) = 1 - \exp\left(-\alpha R_{ijks}(t) - sO_{ijks}(t)\right), \quad \alpha, s \geq 0 \qquad 5$$

where $O_{ijks}(t)$ is the number of observations by i of interactions with option k prior to time t in session s and in all previous sessions and s controls the strength the social learning in a manner analogous to α. This means that inferences regarding s assess the evidence that observation of another individual solving the task exerts a permanent influence on the observer's future rate of interaction with the flap and tube, as oppose to a transient effect (see below). We further generalised learning to investigate the conditions under which direct social learning occurred, by distinguishing different types of observation events, and allowing the rate of social learning to vary between them, i.e.

$$V_{ijks}(t) = 1 - \exp\left(-\alpha R_{ijks}(t) - sO_{ijks}(t)\right)$$
$$where$$
$$s = \begin{pmatrix} s_{All} & s_{AllR} & s_{InR} & s_{InNR} & s_{R} & s_{NR} \end{pmatrix} \qquad 6$$
$$O_{ijks}(t) = \begin{pmatrix} O_{All,ijks}(t) & O_{AllR,ijks}(t) & O_{InR,ijks}(t) & O_{InNR,ijks}(t) & O_{R,ijks}(t) & O_{NR,ijks} & (t) \end{pmatrix}^{T}$$

Here the subscripts All and AllR refer to the observation of manipulations and rewarded manipulations under the assumption that all individuals present during a session saw all manipulations by others in that session[2]. In contrast, subscripts InR, InNR, R and NR refer to cases where the observer was recorded as looking at an individual who was manipulating the task, with InR indicating the observer saw the manipulator both get into the box and obtain food, InNR indicates that the observer saw the manipulator get into the box, but not obtain food, R indicated the observer obtain food, but not get into the box, and NR indicates a

[2] We consider this to be unrealistic, however, if we can show that a putative social learning effect is conditional on apparent observation, it adds considerable support to the evidence.

manipulation was observed, but the observer saw neither box entry or the conspecific gaining food. For example, $O_{InNR,ijks}(t)$ refers to the number of times i was recorded as observing another individual getting into the box, but not recorded as observing it obtaining food[3], and s_{InNR} is a parameter determining the strength of the learning resulting from each of these observations. By testing evidence that each component of s is different to zero and which components are different from other, we can infer what conditions were necessary for social learning to occur, and under which conditions it is strongest.

We found only strong evidence that s_{InR} and s_{InNR} were different to zero, and that there was little evidence of a difference between them (see Model Selection below). We therefore simplified the model to that given in Eqn 6, with $O_{ijks}(t) = O_{InR,ijks}(t) + O_{InNR,ijks}(t)$. We then added in a term to assess the extent to which social learning generalised between the two options:

$$\begin{aligned} V_{ij1s}(t) &= 1 - \exp\left(-\alpha R_{ij1s}(t) - sO_{ij1s}(t) - s_{cross}O_{ij2s}(t)\right) \\ V_{ij2s}(t) &= 1 - \exp\left(-\alpha R_{ij2s}(t) - sO_{ij2s}(t) - s_{cross}O_{ij1s}(t)\right), \end{aligned} \qquad 7$$

where s_{cross} is the permanent effect observation of an individual entering the box through the flap has on the observer's later interactions with the tube, and vice versa. If $s > s_{cross}$, this indicates that direct social learning distinguishes between the two options, whereas if $s = s_{cross}$ it indicates that direct social learning cannot distinguish the two options, perhaps resulting only in a generalised tendency to interact with the box.

Transient Social Effects

We define transient social effects as follows:

1. General Effect (GE): observation of another individual manipulating option k on box l results in a transient increase in the rate of interaction with both options on both boxes.
2. Stimulus enhancement (SE): observation of another individual manipulating option k on box l results in a transient increase in the rate of interaction with option k on both boxes.
3. Box-level local enhancement (BLE): observation of another individual manipulating option k on box l results in a transient increase in the rate of interaction with both options on box l.
4. Specific local enhancement (SLE): observation of another individual manipulating option k on box l results in a transient increase in the rate of interaction with option k on box l.

Any combination of these effects could be occurring, and in principle, they could also be negative (cause a decrease in rate of manipulation). We estimated these in the model by modelling a transient increase in the rate at which an observer manipulated a) the same option (on the same box); b) the different option-type on the same box; c) the same option-type on the different box; and d) the different option-type on the different box. We modelled these

[3] $O_{InR,ijks}(t), O_{InNR,ijks}(t), O_{R,ijks}(t)$ and $O_{NR,ijks}(t)$ were incremented at the end of the relevant observation bout, whereas $O_{All,ijks}(t)$ and $O_{AllR,ijks}(t)$ were incremented at the end of the relevant bout of manipulation.

effects by taking $T_{ijkls}(t)$ to be a function of the time since the times since another individual had last interacted with each option at each box within that session. We assumed that each of these effects would be strongest while a conspecific was interacting with the option in question, and fade away to baseline levels as time went on. We thus modelled each as follows:

$$\begin{aligned} T_{ijkls}(t) &= \theta_{DODB}\exp\left(-\beta x_{ijvws}(t)\right) \\ &+\theta_{SODB}\exp\left(-\beta x_{ijkws}(t)\right) \\ &+\theta_{DOSB}\exp\left(-\beta x_{ijvls}(t)\right) \\ &+\theta_{SOSB}\exp\left(-\beta x_{ijkls}(t)\right) \end{aligned} \quad . \qquad 8$$

v denotes the alternative option-type to *k* and *w* the alternative box to *l*. $x_{ijkls}(t)$ is the time since the last observation of a manipulation by individual i in group j, during session s of option k on box l, excluding manipulations by i, with $x_{ijkls}(0) = \infty$. θ_{DODB} is the effect on the rate of interaction with the different option-type at the different box, etc. $\theta_E \geq 0$ gives the strength of effect E, and $\beta \geq 0$ is the rate at which transient social effects die away, with $H = \ln(2)/\beta$ giving the half-life of the effects. An exponential model seems the natural choice to model an effect that decays to baseline levels as time goes on. This corresponds to a model in which an observer moves into a latent (unobserved) state in which its rate of interaction is raised by θ_E, and which it leaves at constant rate β. Alternatively, this function could be seen as modelling the priming of neural units responsible for task interaction, which fades away over time according to exponential decay. An exponential model also appears to be of an appropriate functional form for the data (see Fig S1).

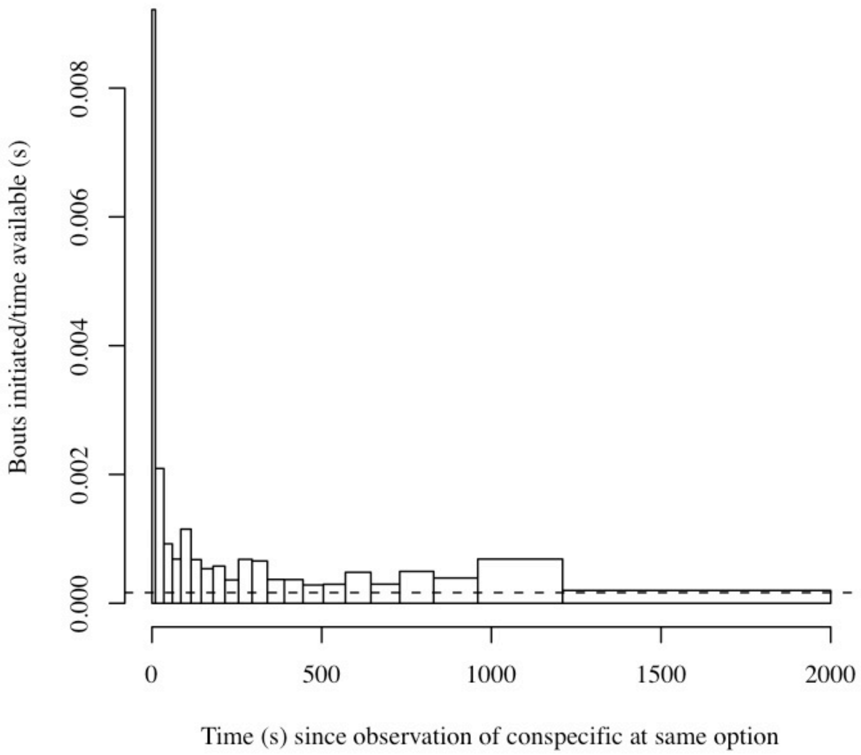

Figure S2. Plot giving an unconstrained estimate of the shape of the transient function for the specific local enhancement effect. Related to Figure 2. This was computed by i) summing, across all individuals, the number of bout initiations within a given time period of observation of a conspecific at that same option; ii) summing, across individuals and options, the total time for which each individual was within a given time period of observation of a conspecific at each option: the 'time available', iii) estimating the rate for each time period by dividing the bouts initiated by the time available. The width of each interval was chosen such that it contained a minimum of 10,000s of time available, with the exception of the final interval, 1210+ s (5029s available time). The dashed line gives the rate before an individual had observed another individual interacting with a given option in that session (886 initiations, 5.4e+06 s available time), taken in the model as infinity.

The transient effects described above were quantified by obtaining the relevant contrasts:

$$\theta_{GE} = \theta_{DODB}$$
$$\theta_{SE} = \theta_{SODB} - \theta_{DODB}$$
$$\theta_{BLE} = \theta_{DOSB} - \theta_{DODB}$$
$$\theta_{SLE} = \theta_{SOSB} - \theta_{BLE} - \theta_{SE} = \theta_{SOSB} - \theta_{DOSB} - \theta_{SODB} + 2\theta_{DODB}\,. \qquad 9$$

The evidence for each (positive) effect is the posterior probability that the relevant contrast is >0. The exception is θ_{GE}, which is constrained to be >0 and was treated as a baseline against which to detect more specific effects. During model selection were found little evidence for

stimulus enhancement, so constrained $\theta_{SODB} = \theta_{DODB}$, i.e. $\theta_{SE} = 0$, before including interaction terms (see below).

Interactions with Individual-Level Variables

The model was expanded to test for differences between different classes of individual in social and asocial learning rates and the strength of transient social effects. This was done one effect at a time by replacing the relevant parameter P with:

$$
\begin{aligned}
&P' \exp(D_{ij}) \\
&\textit{where} \\
&D_{ij} = \gamma_{P,male} male_{ij} + \gamma_{P,pup} pup_{ij} + \gamma_{P,subad} subad_{ij} + \gamma_{P,juv} juv_{ij} + \gamma_{P,dom} dom_{ij} + \varsigma_{P,ij} \, . \\
&\varsigma_{P,ij} \sim N(0, \tau_P)
\end{aligned} \tag{10}
$$

In each case the baseline is a female subordinate adult. Here ς_{ij} is a random effect allowing for individual variation in the parameter P that is not accounted for by the included variables. Without this, we would not take into account sampling error at the level of individuals when estimating the difference between classes. For example, we might happen to have sampled juveniles and sub-adults that are strongly affected by SLE. The random effect takes into account this uncertainty.

Where there appeared to be differences between some age categories but others, we collapsed age categories as appropriate, e.g. including a single effect for juveniles and sub-adults $\gamma_{juvsub} = \gamma_{subad} = \gamma_{juv}$. For the final model, we included any interaction terms for which the 90% central interval did not include zero, and the associated random effects. Where there were no such interactions, we retained the original parameter P in the final model. In the final model we back-transformed the effect for each class of individual, e.g. for the SLE effect for juveniles/sub-adults:

$$\theta_{SLE,juvsub} = \theta_{SLE} \exp(\gamma_{juvsub}(subad_{ij} + juv_{ij}))$$

This gives the median effect for an individual of the relevant class, since the random effect is set to zero.

Derivation of the Log-Likelihood

For each session for each group, the data consists of a series of E_{js} events, which consist of the onsets and offsets of bouts of interacting with a task or observing another individual interacting with the task. This splits each session into $E_{js}+1$ time periods. Each individual either concluded a time period by initiating a bout of interaction with a flap or tube, or it did not. The likelihood for individual i for time period z, starting at time t_z, and concluding at time t_{z+1} with i interacting with option k on box l is therefore:

$L = p($i interacts with option k at box l at $t_{z+1})$ + p(i does not interact with any other options during period z)

And for an individual u who did not interact with any task:

L= p(u does not interact with any options during period z)

Therefore to calculate the likelihood of the data for a given set of parameters, we need the pdf and cdf for the time to interact with an option k at box l from an arbitrary time, t_{start}, for which $x_{ijkls}(t_{start})$ and $V_{ijks}(t_{start})$ are known for all k and l. Since by definition there are no manipulation onset or offset events during period z, we know that $V_{ijks}(t) = V_{ijks}(t_{start})$ for $t_{start} \geq t$. Since there are also no onsets or offsets of bouts of observation, we also know that any individual observing option k at box l immediately after event z will continue to do so for the length of period z, for whom the transient effects corresponding to that observation will remain constant. For such individuals, we only have to increment the rate of manipulation by the appropriate transient effect strengths (θ_E): to simplify the derivation we will ignore these cases for now and add them in later. For all other individuals, the transient effects will decay exponentially to baseline levels meaning that $x_{ijkls}(t_{start} + t) = x_{ijkls}(t_{start}) + t - t_{start}$. This also stands for individuals who have not observed a manipulation on option k on box l, since $x_{ijkls}(t_{start} + t) = x_{ijkls}(t_{start}) = \infty$. We can calculate the required cdf using the following relationship from survival analysis between a hazard function, $\lambda(t)$, and the corresponding cdf, F(t), and pdf f(t)[4]:

$$\lambda(t) = \frac{f(t)}{1-F(t)} \quad \textit{where} \quad f(t) = F'(t) \qquad 11$$

In our case, the hazard function is $\lambda_{ijkls}(t)$. From Eqn. 11 we get:

---

[4] For clarity here we assume $p_{ijs} = 1$ and $f_{ijs}(t) = 1$, i.e. that i is present and free to act, and bring these indicator variables into the likelihood function later.

$$F(t) = 1 - \exp\left(-\int_{t_{start}}^{t} \lambda_{ijkls}(t)dt \right)$$

$$F(t) = \left(1 - \exp\left(-(t - t_{start})\left(\exp\left(\mu_k + I_{ij}\right) + \omega V_{ijks}(t_{start})\right) - \int_{t_{start}}^{t} T_{ijkls}(t) \right)\right). \qquad 12$$

Evaluating the integral, we get:

$$\int_{t_{start}}^{t} T_{ijkls}(t) = \theta_{DODB} \int_{t_{start}}^{t} \exp\left(-\beta\left(x_{ijkws}(t_{start}) + t - t_{start}\right)\right)dt$$

$$+\theta_{SODB} \int_{t_{start}}^{t} \exp\left(-\beta\left(x_{ijkws}(t_{start}) + t - t_{start}\right)\right)dt$$

$$+\theta_{DOSB} \int_{t_{start}}^{t} \exp\left(-\beta\left(x_{ijvls}(t_{start}) + t - t_{start}\right)\right)du$$

$$+\theta_{SOSB} \int_{t_{start}}^{t} \exp\left(-\beta\left(x_{ijkls}(t_{start}) + t - t_{start}\right)\right)dt$$

$$\int_{t_{start}}^{t} T_{ijkls}(t) = -\theta_{DODB}\left(\frac{\exp\left(-\beta\left(x_{ijvws}(t_{start}) + t - t_{start}\right)\right)}{\beta} - \frac{\exp\left(-\beta\left(x_{ijvws}(t_{start})\right)\right)}{\beta} \right)$$

$$-\theta_{SODB}\left(\frac{\exp\left(-\beta\left(x_{ijkws}(t_{start}) + t - t_{start}\right)\right)}{\beta} - \frac{\exp\left(-\beta\left(x_{ijkws}(t_{start})\right)\right)}{\beta} \right)$$

$$-\theta_{DOSB}\left(\frac{\exp\left(-\beta\left(x_{ijvls}(t_{start}) + t - t_{start}\right)\right)}{\beta} - \frac{\exp\left(-\beta\left(x_{ijvls}(t_{start})\right)\right)}{\beta} \right)$$

$$-\theta_{SOSB}\left(\exp\left(-\beta\left(x_{ijkls}(t_{start}) + t - t_{start}\right)\right) - \exp\left(-\beta\left(x_{ijkls}(t_{start})\right)\right)\right)$$

$$= -\frac{1}{\beta}\left(T_{ijkls}(t) - T_{ijkls}(t_{start})\right)$$

which, substituting into Eqn. 12, gives

$$F(t) = \left(1 - \exp\left(-(t - t_{start})\left(\exp\left(\mu_k + I_{ij}\right) + \omega V_{ijks}(t_{start})\right) + \frac{1}{\beta}\left(T_{ijkls}(t) - T_{ijkls}(t_{start})\right) \right)\right), \qquad 13a$$

and differentiating to find the pdf:

$$f(t)=F'(t)=-\begin{pmatrix}-\exp\left(\mu_k+I_{ij}\right)-\omega V_{ijks}(t_{start})\\+\frac{1}{\beta}\left(T_{ijkls}{}'(t)\right)\end{pmatrix}\exp\begin{pmatrix}-(t-t_{start})\left(\exp\left(\mu_k+I_{ij}\right)+\omega V_{ijks}(t_{start})\right)\\+\frac{1}{\beta}\left(T_{ijkls}(t)-T_{ijkls}(t_{start})\right)\end{pmatrix}$$

$$f(t)=\begin{pmatrix}\exp\left(\mu_k+I_{ij}\right)+\omega V_{ijks}(t_{start})\\+T_{ijkls}(t)\end{pmatrix}\exp\begin{pmatrix}-(t-t_{start})\left(\exp\left(\mu_k+I_{ij}\right)+\omega V_{ijks}(t_{start})\right)\\+\frac{1}{\beta}\left(T_{ijkls}(t)-T_{ijkls}(t_{start})\right)\end{pmatrix} \qquad 13b$$

Since:

$$T'_{ijkls}(t)=-\beta\theta_{DODB}\exp\left(-\beta\left(x_{ijvws}(t_{start})+t-t_{start}\right)\right)$$
$$-\beta\theta_{SODB}\exp\left(-\beta\left(x_{ijkws}(t_{start})+t-t_{start}\right)\right)$$
$$-\beta\theta_{DOSB}\exp\left(-\beta\left(x_{ijvws}(t_{start})+t-t_{start}\right)\right)$$
$$-\beta\theta_{SOSB}\exp\left(-\beta\left(x_{ijkls}(t_{start})+t-t_{start}\right)\right)$$
$$T'_{ijkls}(t)=-\beta T_{ijkls}(t)$$

The pdf and cdf can be modified to include individuals who are engaged in an observation during period z (see above) as follows:

$$F(t)=\left(1-\exp\begin{pmatrix}-(t-t_{start})\left(\exp\left(\mu_k+I_{ij}\right)+C_{ijkls}\left(t_{start}\right)+\omega V_{ijks}(t_{start})\right)\\+\frac{1}{\beta}\left(T_{ijkls}(t)-T_{ijkls}(t_{start})\right)\end{pmatrix}\right) \qquad 14a$$

where $C_{ijkls}(t)$ is the effect of any current observations, given by:

$$C_{ijkls}(t)=\theta_{DODB}u_{ijvws}(t)$$
$$+\theta_{SODB}u_{ijkws}(t)$$
$$+\theta_{DOSB}u_{ijvls}(t)$$
$$+\theta_{SOSB}u_{ijkls}(t)$$

where $u_{ijkls}(t)$ is an indicator variable that takes the value 1 if individual i in group j was observing a manipulation on option k on box l during session s at time t, excluding those cases where the observation ended at time t. Note that the relevant parts of $T_{ijkls}(t)$ and $T_{ijkls}(t_{start})$ cancel out in such cases, and so the transient effect for current observations is not counted twice. The pdf now becomes:

$$f(t)=\left(\exp\left(\mu_k+I_{ij}\right)+\omega V_{ijks}(t_{start})+T_{ijkls}(t)\right)\exp\begin{pmatrix}-(t-t_{start})\left(\exp\left(\mu_k+I_{ij}\right)+C_{ijkls}\left(t_{start}\right)+\omega V_{ijks}(t_{start})\right)\\+\frac{1}{\beta}\left(T_{ijkls}(t)-T_{ijkls}(t_{start})\right)\end{pmatrix} \qquad 14b$$

This means the log-Likelihood for the data is:

$$\log(L_{total})=\sum_{j=1}^{G}\sum_{s=1}^{S_j}\sum_{z=1}^{E_{js}+1}\sum_{i=1}^{N_j}p_{ijs}f_{ijs}(t)\left(\log(L_{ijsz})\right), \qquad 15$$

(summing across all groups, sessions, events and individuals) where,

$$\log(L_{ijsz}) = \sum_{l=1}^{2}\sum_{k=1}^{2} y_{ijklsz}\log\Big(\exp\big(\mu_k + I_{ij}\big) + \omega V_{ijks}(t_{js(z-1)}) + T_{ijkls}(t_{jsz})\Big)$$
$$+\sum_{l=1}^{2}\sum_{k=1}^{2}\left(\begin{array}{l} -(t_{jsz} - t_{z-1})\Big(\exp\big(\mu_k + I_{ij}\big) + C_{ijkls}\big(t_{(z-1)}\big) + \omega V_{ijks}(t_{js(z-1)})\Big) \\ +\frac{1}{\beta}\Big(T_{ijkls}(t_{jsz}) - \widehat{T}_{ijkls}(t_{js(z-1)})\Big) \end{array}\right), \qquad 16$$

where y_{ijklsz} is an indicator variable which takes the value 1 if individual i in group j during session s concluded the period z by initiating a bout of manipulation of option k on box l; all $t_{js0} = 0$; $t_{jsE_{js}}$ is the time session s concluded for group j; t_{jsz} is the time of the zth event in session s for group j for $0 < z < E_{js}$. $\widehat{T}_{ijkls}(t_{js(z-1)})$ gives the transient effect evaluated immediately after the (z-1)th event and $T_{ijkls}(t_{jsz})$ gives the transient effect evaluated immediately prior to the zth event.[5]

---

[5] Some computational difficulties were encountered when β was close to zero: therefore when β was equal to zero to the accuracy used by WinBUGS we used the likelihood function

$$\log(L_{ijsz}) = \sum_{l=1}^{2}\sum_{k=1}^{2} y_{ijklsz}\log\Big(\exp\big(\mu_k + I_{ij}\big) + \omega V_{ijks}(t_{js(z-1)}) + T_{ijkls}(t_{jsz})\Big)$$
$$+\sum_{l=1}^{2}\sum_{k=1}^{2}\Big(-(t_{jsz} - t_{z-1})\Big(\exp\big(\mu_k + I_{ij}\big) + \widehat{T}_{ijkls}(t_{js(z-1)}) + \omega V_{ijks}(t_{js(z-1)})\Big)\Big),$$

which corresponds to a transient effect that operates at full strength for the remainder of the session.

Markov Chain Monte Carlo (MCMC)

We used MCMC to generate samples from the posterior distribution for the parameters in the model, using WinBUGS 1.4 [35], which we analysed using the CODA package [36] in the R statstical environment [37]. This approach was chosen in preference to maximum likelihood, since it is relatively easy to include random effects and the posterior distribution for each parameter is automatically averaged over uncertainty in the other parameters in the model [41]. We used the "zeroes-ones trick" to specify the likelihood for the model [42] (p.276).

For each model, we ran 3 chains with different starting values. We ran and discarded a burn in period of at least 4000 iterations, using the Gelman Rubin statistic and time series of the model parameter values to check for convergence. We ran at least 33,334 further iterations for each chain to ensure we had an effective posterior sample of 1000 for all parameters after allowing for autocorrelations. For estimates of parameters and contrasts we used the median of the posterior sample, which minimises the absolute error and is invariant to monotonic transformations [43]. For 95% credible intervals we use highest posterior density (HPD) intervals.

Prior distributions were chosen to be low-information priors [42]. We used a non-informative prior when these were not improper distributions, i.e. for parameters bounded between 0 and 1: s and α parameters, we used prior ~U(0, 1). For variance parameters, τ_{ind} and τ_{group}, we used a gamma distribution, with shape=1 and rate=0.1. For other parameters constrained to be greater than zero, we set log(ω)~N(0, 0.001), log(β)~N(0, 0.01) and log(θ_E)~N(0,0.001) for all E, and for unbounded parameters, μ,κ and γ parameters, we set the prior ~N(0, 0.001). These were selected to be as uninformative as possible without causing numerical errors.

Model selection procedure

In general, for any parameters that were not bound above zero, or contrast between parameters, we examined whether their 90% central interval (5th-95th percentile) included zero. If it did then we excluded the parameter, or, for a contrast, constrained the relevant parameters to be equal. For parameters constrained to be greater than zero, such as s parameters, we fit an alternative model in which the parameter was constrained to zero, and favoured the model with the lower deviance information criterion (DIC).

We initially fit a model with all individual level variables (Eqn. 2), all transient social effects (Eqn 8) and a single parameter s for direct social learning (Eqn. 5), with no interactions between individual-level variables and other variables. We dropped all individual level variables not significantly different to zero: here we found only $\kappa_{male} > 0$, indicating evidence that males tended to have a higher baseline rate of interaction than females.

We then tested for differences in the effects of direct social learning resulting from different classes of observation, using Eqn. 6. We found that the estimates for s_{All} and s_{AllR} were very small (see Fig S2) and significantly smaller than the estimates for individuals having been recorded as observing the event. We found no significant difference between cases where the observer saw a conspecific enter the box and gain a reward, and seeing it enter the box but not observe reward (95% central interval $s_{InR} - s_{InNR}$: [-0.0047,0.0041]), so we set

$s_{InR} = s_{InNR} = s_{In}$. We then found that observations of a conspecific entering the box had a greater effect than observations not including box entry, but including observation of a conspecific being rewarded (95% central interval $s_{In} - s_R$: [0.00030,0.0048]). We found there were very few observations (twenty) where the observer neither saw a conspecific being rewarded or entering the box, so the effects of this class of observation could not be estimated with any precision. We therefore fit another model, with $s_{NR} = s_R = s_{NotIn}$, and found that observation of an individual entering the box resulted in a greater effect than observation that did not include box entry (95% central interval $s_{In} - s_{NotIn}$: [0.00029,0.0048]). We therefore retained only term $s = s_{In}$, which gives us the model in Eqn. 5 with $O_{ijks}(t) = O_{InR,ijks}(t) + O_{InNR,ijks}(t)$. Setting s=0 resulted in an increase in DIC, so this term was retained in the model. Taken together these results indicate evidence of a direct social learning effect that is conditional on observation of a conspecific gaining entry to the box, and results in a higher rate of interaction with the specific option (flap or tube) used by the demonstrator.

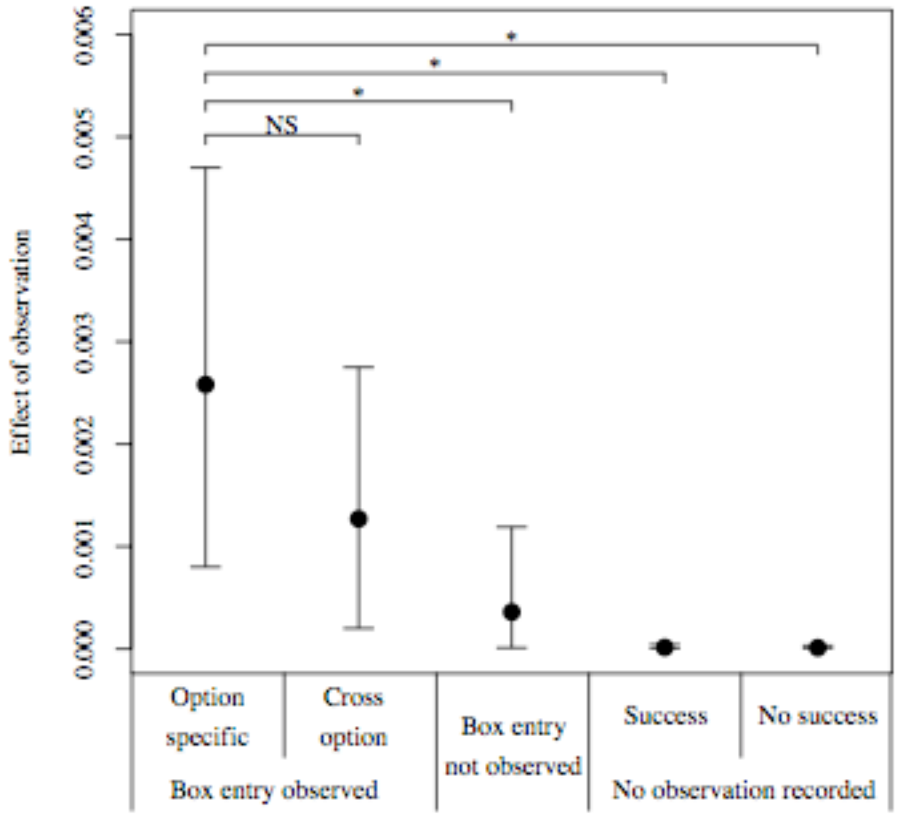

Figure S1 Mean and 95% central interval of the posterior distribution for the direct social learning effect of different classes of observation. The size of each effect refers to the corresponding parameter in the learning rule used in the model (Eqn. 6, with constraints $s_{InR} = s_{InNR}$ and $s_R = s_{NR}$). * indicates that the 95% central interval for the contrast between two effects did not include zero; NS indicates that the 95% central interval for the contrast between two effects did include zero. Note that only the effects for "Box entry observed" were retained in the final model.

We then tested whether the effect generalised to the option not used by the observed conspecific, by expanding the model as shown in Eqn. 7. We found that the 95% central interval for $s - s_{cross}$ included zero and inclusion of s_{cross} in the model caused a decrease in DIC, indicating there is not strong evidence that the effect is option specific. In summary, there is evidence of direct social learning, but there is only weak evidence that this effect is option specific.

We found evidence for a positive effect of SLE (95% central interval= [0.0021, 0.0049]) and BLE (95% central interval= [3.0E-4, 0.0015]), but little evidence for a positive effect of SE (95% central interval= [-2.2E-4, 5.0E-4]), so we dropped the latter from the model as described above. We then fitted alternative models on which the transient effects were conditional on i) observation of a conspecific gaining entry to the box, ii) observation of a conspecific obtaining a reward or iii) both. We also fitted a model in which the transient effects operated on all individuals present at an experimental session. In all cases the DIC was much higher (>100) than that in the original model indicating evidence that observation of interaction with an option was a necessary and sufficient condition for the transient social effects to occur.

Next we tested for significant interactions of individual level variables with the rate of asocial learning, α, finding evidence only that dominant adults had a lower rate of asocial learning than other individuals. This indicates that dominants' future rate of interaction with an option-type was less affected by a successful manipulation of that option. Next we tested for interactions with the rate of direct social learning, s, finding no strong evidence of any differences between classes of individuals. Finally, we tested for interactions with the transient social learning effects, i.e. θ parameters. We found evidence that pups, juveniles and sub-adults were more affected by specific local enhancement than adults (interaction with θ_{SOSB}), with juvenile and sub-adults being more strongly affected than pups, but that there was no significant difference between juveniles and sub-adults. We also found evidence that the general effect was significantly weaker in juveniles and sub-adults (interaction with θ_{DODB}). Taken together, these results suggest that the transient social effect is strongest and most spatially specific for juveniles and sub-adults, then pups, with adults being the least affected, and with the least spatial specificity. This leaves us with the following final model:

$$\lambda_{ijkls}(t) = \left(\exp\left(\mu_k + \kappa_{male} male_{ij} + \eta_{ij} + \nu_j\right) + \omega V_{ijks}(t) + T_{ijkls}(t)\right) p_{ijs} f_{ijs}(t)$$

where

$$V_{ij1s}(t) = 1 - \exp\left(\alpha \exp\left(\gamma_{\alpha,dom}(dom_{ij}) + \varsigma_{\alpha,ij}\right) R_{ijks}(t) - sO_{ij1s}(t) - s_{cross} O_{ij2s}(t)\right)$$

$$V_{ij2s}(t) = 1 - \exp\left(\alpha \exp\left(\gamma_{\alpha,dom}(dom_{ij}) + \varsigma_{\alpha,ij}\right) R_{ijks}(t) - sO_{ij2s}(t) - s_{cross} O_{ij1s}(t)\right)$$

$$O_{ijks}(t) = O_{InR,ijks}(t) + O_{InNR,ijks}(t)$$

and

$$T_{ijkls}(t) = \theta_{DODB} \exp\left(\gamma_{DODB,juvsub}\left(juv_{ij} + subad_{ij}\right) + \varsigma_{DODB,ij}\right) \sum_q \exp\left(-\beta x_{ijqws}(t)\right)$$

$$+\theta_{DOSB} \exp\left(-\beta x_{ijvls}(t)\right)$$

$$+\theta_{SOSB} \exp\left(\gamma_{SODB,juvsub}\left(juv_{ij} + subad_{ij}\right) + \gamma_{SODB,pup} pup_{ij} + \varsigma_{SOSB,ij}\right) \exp\left(-\beta x_{ijkls}(t)\right)$$

17

We generated 95% credible intervals for each parameter, and, contrast (see Table S1). We tested again for differences in the rate of direct social learning, in the context of the final model, which indicated again evidence that observation of a conspecific gaining entry to the box was necessary for direct social learning to occur (see Fig S2). We also tested again for transient effects conditional on observation of a conspecific gaining entry to the box (ΔDIC= +168.2), observation of a conspecific obtaining a reward (ΔDIC= +137.8). We also fitted a model in which the transient effects operated on all individuals present at an experimental session (ΔDIC= +399.2). Taken together, these findings indicate evidence that observation of interaction with an option was a necessary and sufficient condition for the transient social effects to occur.

In light of the findings of the analysis concerning the rate of solving and task abandonment (see below), we also tested for an effect of previous unsuccessful interactions with a specific option-type on the rate of interaction with the same option-type, by including this as a term in the linear predictor. Surprisingly, this yielded a positive effect (95% central interval= [0.063, 0.219], which *prima facie* appears to contradict the principles of reinforcement learning, with unrewarded task manipulations causing an increase in the rate of future manipulations. However, an alternative explanation is that individuals who were highly motivated to interact with the task also accrued more unsuccessful manipulations. To distinguish between these explanations, we created a new variable by subtracting the mean number of previous unsuccessful manipulations across the diffusion, for each individual. The effects of this new variable are now only likely to reflect changes caused by differences in the number of unsuccessful attempts *within* each individual. These were estimated to have a negative effect (95% central interval= [-0.392, -0.080]) as is consistent with reinforcement learning. However the inclusion of the effect did not improve the model (ΔDIC= +1.5), so we retained the final model given in Eqn. 17.

Causal interpretation of the model

The results of the model indicate a statistical pattern that is consistent with a specific local enhancement effect that is stronger in juveniles, sub-adults and pups, and a direct social learning effect of observation. Since these patterns are based on observation rather than direct experimental manipulation of observational experiences, we cannot completely rule out the possibility that an unknown variable is influencing manipulation and observation of the task in a way that results in an identical statistical pattern. However, the specificity of each effect (spatial specificity of the transient effect and conditionality of the direct effect on observation of another individual gaining entry to the box) rules out most alternative explanations for the observed patters. Here we present further analyses that rule out what we consider to be the most plausible alternative explanations.

One possibility is the apparent direct effect of observation is caused by individual variation in the time spent close to the task. Individuals who spend a lot of time close to the task are more likely to observe manipulations, and are more likely to interact with the task, even if the former does not cause the latter. The fact that individuals had to see a conspecific gain entry to the box for the effect to occur goes someway to weakening this alternative explanation. In addition, we would not expect this alternative process to result in a cumulative increase in the rate of interaction, as we would with direct social learning. Rather we would expect the total number of observations made by each individual to predict the time spent near the box, and consequently the rate of manipulation. Therefore, we controlled statistically for this possibility by including the total number of observations made by each individual (corrected for the number of sessions present) as an individual-level variable in I_{ij}. We found that even with this control variable in the model, setting s=0 resulted in a large increase in DIC (+12.4), indicating strong evidence for directed social learning. However, setting $s_{cross} = 0$ now resulted in a large decrease in DIC (-15.3), and the resulting model also had a lower DIC than the final model given above (-9.4). This suggests the apparent cross-option generalisation of direct social learning may be an artefact of this non-causal process.

An alternative explanation for the apparent transient effect of observation is that individuals who happened to be close to a specific option on a specific box were both more likely to observe individuals interacting with it, and more likely to interact with it themselves, without the former causing the latter. Again, the fact that the strength of the effect differs greatly between classes of individuals goes some way to weakening this alternative explanation. In addition, if the alternative explanation is true, we would expect an observer to be more likely to choose the option it is closest to at the end of an observation, rather than the option it saw a conspecific interacting with[6].

We tested this alternative hypothesis by reanalysing the videos for a random two sessions for each group. For each observation event, we recorded, to the nearest 5cm, how close the observer was to the demonstrated option at the end of the observation bout, and how close it was to the nearest non-demonstrated option. We then recorded which option, if any, was chosen next by that individual in the same session and before its next observation event. This yielded 65 observations by 34 individuals that were followed by a choice. In 29 of these, the closest non-demonstrated option was closer than the demonstrated option, yet in these cases

[6] Though this might still be the result of a causal effect, if observers moved closer to the option being demonstrated as a result of observation, rather than being more likely to observe demonstrations at the option that happened to be closest.

the demonstrated option was chosen 15 times (52%), the closest option 4 times (14%) and one of the other options 10 times (35%). This means that the demonstrated option was chosen more often than we would expect if an option was chosen at random (binomial test: expected proportion=0.25; $p<0.001$) and was chosen significantly more often than the closest option (binomial test: expected proportion=0.5; $p<0.001$). The effect is stronger if we only examine choices made within 60s of observation: 14/24 demonstrated; 3/24 closest; 7/24 other. Demonstrated versus non-demonstrated, Binomial test: expected proportion=0.25; $p<0.001$; demonstrated versus closest, binomial test: expected proportion=0.25; $p<0.001$. Therefore the data point to the fact that the demonstrated option is more likely to be chosen, even when it is not the closest.

In conclusion, the data do not seem consistent with the alternative explanations considered above, and we consider causal social influences to be the most likely explanation for the observed statistical patterns.

3. Modelling probability of observation

For each individual, we calculated the total number of manipulation events made by other individuals during each session in which it was present, and the number of these that the individual was recorded as observing. We then fitted a generalised linear mixed model with a binomial error structure and logit link function, with individual and group as nested random effects to model the probability that an individual would observe any given manipulation event. We used MCMC to generate samples from the posterior distribution for the parameters in the model, using WinBUGS 1.4 [35]. We tested for differences in the probability of observation between a) males and females, and b) pups, juveniles, sub-adults, subordinate adults and dominant adults by examining whether the 95% central intervals for the difference between classes of individuals included zero. The only effect for which the 95% credible intervals did not include zero was that pups were more likely to observe a manipulation than subordinate adults.

4. Modelling probability of successful manipulation

To model the probability that an individual would be successful (i.e. obtain food) in a given bout of manipulation with the task we used a GLMM with a binomial error structure and logit link function, with nested random effects for group and individual. We allowed for a difference in difficulty between flap and tube and tested for between-individual differences in the probability of success between males and females, pups, juveniles, sub-adults, subordinate adults and dominant adults. We also tested for how probability of success depended on an individual's prior experience. As before, we assumed that potential influences could be *a*) an individual's own history of manipulations, i.e. the cumulative number of successful interactions and number of unsuccessful interactions at the option being manipulated; *b*) direct social learning: a permanent effect resulting from observation, i.e. the cumulative number of observed successful manipulations at each option, and *c*) transient social influence, i.e. the time since another individual last interacted with the same option at the same box[7]. The initial full model had a linear predictor, ψ_{ijkls}:

$$\psi_{ij1ls} = \mu + B_{ij} + \eta_{time}s + \eta_{AL+}R_{ij1s}(t) + \eta_{AL-}U_{ij1s}(t)$$
$$+\eta_{NSSL}\left(O_{ij1s}(t) + O_{ij2s}(t)\right) + \eta_{OSSL}O_{ij2s}(t) + g(x_{ij1ls}(t))$$
$$and$$
$$\psi_{ij2ls} = \mu + \eta + B_{ij} + \eta_{time}s + \eta_{AL+}R_{ij2s}(t) + \eta_{AL-}U_{ij2s}(t)$$
$$+\eta_{NSSL}\left(O_{ij1s}(t) + O_{ij2s}(t)\right) + \eta_{OSSL}O_{ij1s}(t) + g(x_{ij2ls}(t))$$
$$where$$
$$B_{ij} = \eta_{male}male_{ij} + \eta_{pup}pup_{ij} + \eta_{subad}subad_{ij} + \eta_{juv}juv_{ij} + \eta_{dom}dom_{ij} + \eta_{ij} + \nu_j$$
$$\eta_{ij} \sim N\left(0,\tau_{ind}\right)$$
$$\nu_j \sim N\left(0,\tau_{group}\right)$$

where $\log it\left(p_{ijkls}(t)\right) = \psi_{ijkls}$ gives the probability, $p_{ijkls}(t)$, of individual *i* in group *j* succeeding in a bout of interaction with option *k* on box *l*, commencing at time *t* during session *s*; μ is the coefficient, η_{tube} allows for underlying differences between options; η_{male}, η_{pup}, η_{subad}, η_{juv} and η_{dom} give the effects of the individual variables; η_{time} models and controls for systematic time effects on the probability of solving, across sessions; η_{AL+} gives the effect of a previous successful interaction on a specific option, with $R_{ijks}(t)$ giving the number of successful interaction with option *k* made by *i* prior to time *t* in session *s* and in previous sessions; η_{AL-} gives the effect of a previous unsuccessful interactions on a specific option, with $U_{ijks}(t)$ giving the number of unsuccessful interactions with option *k* made by *i* prior to time *t* in session *s* and in previous sessions; η_{NSSL} gives the option non-specific effect of social learning, i.e. the effect observation of a successful manipulation has on the probability of success of either option, whereas η_{OSSL} gives the option-specific effect of social learning, over and above that of the non-specific effect; $O_{ijks}(t)$ gives the number of successful interactions with option *k* observed by *i* prior to time *t* in session *s* and in previous sessions (entry to the box observed).

---

[7] This particular transient effect was chosen as the most likely to be in operation in light of its dominant effect on the rate of interaction.

$g(x_{ijkls}(t))$ is a function of the time since an individual last interacted with the same option on the same box. Initially we modelled this as a linear function but found no significant effect, and then as a step function:

$$g(x_{ijkls}(t)) = \begin{cases} \eta_{trans} & x_{ijkls}(t) \leq T_{trans} \\ 0 & x_{ijkls}(t) > T_{trans} \end{cases}, \qquad 19$$

where η_{trans} is the transient effect of another individual at the option in question, and T_{trans} determines how long the effect lasts. We tried a number of values for T_{trans}, but the strongest effect was found for T_{trans}=0, with η_{trans} <0, indicating only that an individual was less likely to solve the task if another individual was present at the chosen option/box at the onset of manipulation.

The model was fitted using the lmer function in the lme4 package [39] of the R statistical environment [37], using the Laplace approximation.

Instead of using a model selection procedure to choose a best model, we used a model averaging approach, using AIC (Akaike's Information Criterion) [15]. Inferences based on model averaging take into account our uncertainty as to which is the best model. AIC estimates the Kullback-Leibler (K-L) information for a model: the extent to which the predicted distribution for the dependent variable approximates its true distribution (lower values better). This allows us to calculate an Akaike weight for each model, which gives the likelihood the model is the actual best K-L model (that with the lowest K-L information) out of those considered, allowing for sampling variability. Therefore, by summing the Akaike weights for all models including a specific variable, we obtain the probability that that variable is in the best K-L model, thus quantifying the support the data give for an effect of that variable [15].

We fitted models including every combination of fixed effects shown in Eqn. 18, and models with the constraints, with random effects retained in all models. This was done automatically using R code that fitted each model, and recorded the AIC, model coefficients and standard errors in each case. We also varied the conditions under which direct social learning was assumed to occur with *i*) all observations resulting in learning; *ii*) only observations of an individual being rewarded resulting in learning; *iii*) only observations of an individual gaining entry to the box resulting in learning; or *iv*) only observations of an individuals gaining entry to the box and being rewarded resulting in learning. We also considered models in which the effects of direct social learning were a) option specific or b) option general (see Eqn. 18). To evaluate the support for each model of social learning, we summed the Akaike weights for each of combination of i-iv and a/b as well as for models with no direct social learning, to calculate the support for each. Where presence versus absence of a variable is concerned, the total Akaike weight gives the support for its inclusion in the K-L best model with >50% indicating more support for than against its inclusion. Where multiple mutually exclusive "sub-models" are considered for a variable (see Tables S2-7) one must instead compare the Akaike weights for each proposed sub-model, and that for models in which the variable is absent. The relative size of the Akaike weights then give the relative support for each sub-model.

We present model-averaged estimates of the effect of each variable for which there is reasonable support, conditional on its presence in the model, and Wald 95% confidence intervals based on the unconditional standard error, which allows for model selection uncertainty [15]. We present these on the scale of the linear predictor, i.e. the effect on the natural logarithm of the odds of success. We feel that this approach is preferable to calculating a p-value to quantify the strength of evidence for each effect, since a) the p value depends on which model is chosen, and so does not account for model uncertainty, and b) a large p value tells us little about the strength of evidence against the effect (whilst statistical power can be calculated, this has to be for a specified, usually arbitrary, effect size [44].

Contrary to expectations, there was strong support for a negative effect of the number of past successes at the task, and this effect was not option specific. This is surprising since we would expect individuals who solve the task to be more likely to do so in the future. Consequently we decided to investigate this further. To be sure that we were testing for a within-individual effect we centred the number of past successes and failures by subtracting the average across all bouts of interaction for the individual in question, i.e.

$$\begin{aligned} R'_{ijks}(t) &= R_{ijks}(t) - \mu_{R,ijk} \\ U'_{ijks}(t) &= U_{ijks}(t) - \mu_{U,ijk} \end{aligned}, \qquad 20$$

where $\mu_{R,ijk}$ is the average number of previous successes across bouts of interaction with option k by individual i in group j, and $\mu_{U,ijk}$ is the average number of previous unsuccessful interaction with option k by i in j. We also formulated another variable:

$$S_{OS,ijks}(t) = \begin{cases} 1 & R_{ijks}(t) > 0 \\ 0 & R_{ijks}(t) = 0 \end{cases}, \qquad 21a$$

such that $S_{OS,ijks}(t)$ indicated whether an individual had previously solved the task using option-type *k* (OS denotes option-type specific), and

$$S_{NS,ijs}(t) = \begin{cases} 1 & R_{ij1s}(t) + R_{ij2s}(t) > 0 \\ 0 & R_{ij1s}(t) + R_{ij2s}(t) = 0 \end{cases}, \qquad 21b$$

such that $S_{NS,ijs}(t)$ indicated whether an individual had previously solved the task using either option-type (NS denotes option non-specific). We then updated Eqn. 18 as follows:

$$
\begin{aligned}
&\psi_{ij1ls} = \mu + B_{ij} + \eta_{time}s + \eta_{AL+}R'_{ij1s}(t) + \eta_{AL-}U'_{ij1s}(t) \\
&+\eta_{Solved(OS)}S_{OS,ij1s}(t) + \eta_{Solved(NS)}S_{NS,ijs}(t) \\
&+\eta_{NSSL}\left(O_{ij1s}(t) + O_{ij2s}(t)\right) + \eta_{OSSL}O_{ij1s}(t) + g(x_{ij1ls}(t)) \\
&\textit{and} \\
&\psi_{ij2ls} = \mu + \eta_{tube} + B_{ij} + \eta_{time}s + \eta_{AL+}R'_{ij2s}(t) + \eta_{AL-}U'_{ij2s}(t) \\
&+\eta_{Solved(OS)}S_{OS,ij2s}(t) + \eta_{Solved(NS)}S_{NS,ijs}(t) \\
&+\eta_{NSSL}\left(O_{ij1s}(t) + O_{ij2s}(t)\right) + \eta_{OSSL}O_{ij2s}(t) + g(x_{ij2ls}(t)) \\
&\textit{where} \\
&B_{ij} = \eta_{pup}pup_{ij} + \eta_{subad}subad_{ij} + \eta_{juv}juv_{ij} + \eta_{ij} + \nu_j \\
&\eta_{ij} \sim N(0,\tau_{ind}) \\
&\nu_j \sim N(0,\tau_{group})
\end{aligned}
, \qquad (22)
$$

where $\eta_{Solved(NS)}$ gives the effect of previously having solved the task using either option-type, and $\eta_{Solved(OS)}$ gives the additional effect of previously having solved the task using the same option-type. We dropped the non-significant effects of sex and dominance, in order to reduce the models to be fitted to a manageable number. We then used the used the same model selection procedure as before, but also fitted alternative models in which the effect of the number of previous manipulations was not option specific, i.e. replacing each of the terms: $+\eta_{AL+}R'_{ij1s}(t) + \eta_{AL-}U'_{ij1s}(t)$ and $+\eta_{AL+}R'_{ij2s}(t) + \eta_{AL-}U'_{ij2s}(t)$
with
$+\eta_{AL+}\left(R'_{ij1s}(t) + R'_{ij2s}(t)\right) + \eta_{AL-}\left(U'_{ij1s}(t) + U'_{ij2s}(t)\right)$.

There was strong support for models in which there was an option specific effect of having solved the task before (total Akaike weight= 92.1%), with little support for models in which there was a cross-option effect (total Akaike weight= 32.6%). The log odds of success were higher for those who had solved the task using that option type before (+0.23 [0.0034, 0.46]). However, there was strong evidence of an additional negative effect of further number of successes and a positive effect of the previous number of failures (see Table S4). The data indicate that this was more likely to be an option-general effect. These effects were estimated at -1.7 [-3.0, -0.44] per previous success and 0.34 [0.14,0.55] per previous failure.

	Total Akaike weight (%)	
No effect	0	
	Option-specific	Option-general
Failures only	0	0
Successes only	0.3	0.4
Both with same effect	0.1	0.1
Both with different effect	26.8	72.4

Table S4. Relative support for different models of the effect of previous number of successes and/or failure on probability of success.

With regards to direct social learning, there was strongest support for models in which both observation of box entry and reward were necessary for social learning to occur and that its effects were option general (see Table S5). The only other model with comparable support was one in which observation of reward was sufficient for social learning to occur. We conclude that it is highly probable direct social learning affected the probability of success, and that observation of reward was a necessary condition for this to occur. Furthermore, it is likely that observation of box entry was also a necessary condition. Conditional on the most supported model of social learning, but averaged across all other models considered, we estimate the effect per observation to be 0.30 (95% CI= [0.090,0.50]) on the log odds scale.

	Total Akaike weight (%)	
No social learning	1.2	
Conditions for social learning:	Option-specific	Option-general
All observations	1.3	2.4
Observations of reward	0.7	20.4
Observations of box entry	0.7	0.6
Observations of both box entry and reward	4.2	68.7

Table S5. Relative support for different models of the effect of direct social learning on probability of success.

There was strong support for an effect of day (total Akaike weight= 97.5%), however, its effect was not estimated with great precision (-0.14 [-0.48,0.20] per day) since its effects varied greatly depending on which other variables were included in the model. There was strong support for differences between age categories (total Akaike weight= 91.9%). The baseline (adults) was estimated at -2.3 [-3.6, -1.0] with differences relative to adults of -0.93 [-1.8,-0.042] for pups; -0.84 [-3.5,1.8] for juveniles; and -0.66 [-1.5,0.17] for sub-adults. There was not strong support for an effect of another individual's presence at the option being manipulated (total Akaike weight= 28.0%), but we cannot rule out a difference between option-types (total Akaike weight= 50.5%).

Changes in the probability of success in a bout could logically be the result of only two factors: *a*) changes in the rate at which individuals terminate a bout of interaction unsuccessfully, henceforth 'task abandonment'; or *b*) changes in the rate at which individual terminate a bout successfully, henceforth 'rate of solving'. To investigate how each variable operated, we fitted a separate model of each process, using a Cox Proportional Hazards survival analysis model [14]. For *a*), the time of 'death' is the time since initiating a bout at which an individual terminates a bout without gaining a reward. Those individuals who gain a reward are considered to be 'censored', equivalent to surviving the course of a survival analysis. Conversely, for *b*) the time of 'death' is the time since initiating a bout at which an individual terminates a bout by gaining a reward. In this case, those individuals who do not gain a reward are 'censored'. The models were fitted using the coxme function in the coxme package [40] in the R statistical environment [37]. We fitted models according to the linear predictor given in Eqn. 22 above, excluding the coefficient, μ, which is not necessary in a Cox Proportional Hazards model, and the dependent variable being time since the start of the bout of interaction. For each model we used the same model averaging procedure as that

described above, calculating AIC using the integrated likelihood. However, here we report effects as the back-transformed multiplicative effects on rate.

In agreement with the previous model, there was some evidence that meerkats who had previously solved the task using a specific option solved at a faster rate (x1.51 [1.00, 2.01]) when manipulating the same option type (total Akaike weight= 68.2%), but little evidence that this generalised to the other option type (total Akaike weight= 37.4%). There was little support for an effect of further manipulations, but weak evidence for a positive effect of previous number of unsuccessful manipulations (x1.12 [1.01, 1.25] per manipulation), which is more likely to have been option type specific than option type general (see Table S6).

	Total Akaike weight (%)	
No effect	3.3	
	Option-specific	Option-general
Failures only	37.0	13.1
Successes only	2.7	2.9
Both with same effect	4.6	4.1
Both with different effect	21.7	10.7

Table S6. Relative support for different models of the effect of previous number of successes and/or failure on rate of solving.

There was little support for models including a difference between option-types (total Akaike weight= 28.4%), an effect of session (total Akaike weight= 36.6%) or an effect of other individuals' presence at the option being manipulated (total Akaike weight= 28.7%). There was reasonable support for models including difference in rate of solving between age categories (total Akaike weight= 79.7%). These effects were estimated as (relative to adults): x0.68 [0.42, 1.11] for pups; x0.57 [0.34,0.97] for juveniles; and x0.78 [0.37, 1.6] for sub-adults.

There was not conclusive evidence for or against direct social learning. Models in which observations of both box entry and reward resulted in option-type general learning were again most favoured, but the level of support was only 1.34x greater than that for models with no direct social learning (see Table S2). We conclude that there is not strong evidence that direct social learning influences the rate of solving, but we cannot rule out the possibility of an option-type general effect.

	Total Akaike weight (%)	
No social learning	18.2	
Conditions for social learning:	Option-specific	Option-general
All observations	8.6	8.1
Observations of reward	7.6	9.9
Observations of box entry	7.2	8.4
Observations of both box entry and reward	7.2	24.5

Table S2. Relative support for different models of the effect of direct social learning on rate of solving.

In contrast, there was strong evidence for direct social learning affecting task abandonment, and that this effect was option-type general and required observation of both box entry and reward (see Table S3). The effect was estimated at x0.84 [0.76, 0.94] per observation.

	Total Akaike weight (%)	
No social learning	0.8	
Conditions for social learning:	Option-specific	Option-general
All observations	0.5	1.2
Observations of reward	0.4	3.9
Observations of box entry	0.4	0.6
Observations of both box entry and reward	5.9	86.3

Table S3. Relative support for different models of the effect of direct social learning on rate of task abandonment.

In agreement with the analysis of probability of success, there was strong support for an option-type specific effect of having solved the task previously (total Akaike weight= 91.7%) but equivocal support for on option-type general effect (total Akaike weight= 54.3%). The rate of task abandonment was lower for those who had solved the task using that option type before (x0.34 [0.23, 0.49]). However, there was strong evidence of an additional positive effect of further number of successes and a negative effect of the previous number of failures (see Table S7). The data indicate that this was more likely to be an option-general effect. These effects were estimated at x1.09 [1.04, 1.14] per previous success and x0.84 [0.74,0.96] per previous failure.

	Total Akaike weight (%)	
No effect	0.1	
	Option-specific	Option-general
Failures only	0	0
Successes only	4.3	1.4
Both with same effect	0.5	0.7
Both with different effect	16.5	76.4

Table S7. Relative support for different models of the effect of previous number of successes and/or failure on rate of task abandonment.

There was not strong support for differences between age categories (total Akaike weight= 55.5%) or a effect of the presence of another individual at the same specific option (total Akaike weight= 32.8%). There was weak evidence of a difference between option-types (total Akaike weight= 79.7%), with the tube having a lower rate of abandonment than the flap (x0.76 [0.55, 1.06]). There was also evidence that the rate of task abandonment decreased over sessions (total Akaike weight= 88.4%; x0.91 [0.83,1.00] per day), which indicates a time effect not accounted for by the learning effects included in the model.

For model diagnostics, we examined plots of residuals from a coxph model (survival package [45] fit with an equivalent fixed effects structure to the best model found, with individual included as a random effect (only a single random effect is allowed by coxph, but coxme does not allow extraction of residuals). We used 'dfbeta' residuals to assess the influence of each observation, which estimate the effect on each parameter of dropping the observation from the model. No observations had a large influence on the parameters in the final models. We used plots of martingale residuals against each covariate to assess linearity, which was found to be appropriate in each case. We checked the assumption of proportional hazards by using the cox.zph function to test for a correlation between the scaled Schoenfeld residuals, for each effect in the final model, and time. These were found to be non-significant for all effects on the rate of solving. However, for task abandonment, a significant positive correlation was found for all coefficients of the age category factor, and a negative correlation for the effect of the number of previous observations (direct social learning). Plots of the scaled Schoenfeld residuals suggested that these effects tended to become stronger as time (from the start of interaction) went on. Details of these model diagnostic tests can be found in [46].

In summary, most of the variables influencing the probability an individual will solve the task during a bout of interaction operate by influencing the rate at which individuals terminate a bout of interaction unsuccessfully. There is strong evidence for direct social learning influencing the *probability* of solving, which persists after motivational and asocial learning variables have been accounted for. There is not strong evidence that direct social learning influences the *rate* at which individuals solve the task, nor that its effects are specific to the option-type chosen to solve the task, suggesting that observers do not learn the motor pattern required to solve the task, through imitation or emulation. Instead the evidence suggests that

social learning decreases the rate at which individuals abandon the task once they have started to interact with it.

5. Modified Option Bias method

The Option Bias method [19] is a statistical test for detecting whether individuals within a group are more likely to use the same option to solve a task than would be expected by chance and asocial learning alone. This is done by applying a conventional statistical test to test for a group-level bias that assumes manipulations by the same individual are uncorrelated, and using this to yield a test statistic. In this case we used a GLM with a binomial error structure, with the choice of tube/flap as the binary response variable, and group as the explanatory variable, using the LRT statistic as the test statistic. In the Option Bias test the null distribution is generated by randomising individuals between groups. However, in this case the presence of demonstrators trained to use different options in different groups artificially inflates the option bias effect. Therefore we modified the randomisation procedure so that demonstrators were constrained to remain in different groups.

6. Comparison with Thornton & Malapert [20]

Despite strong evidence that social learning influenced the meerkats in this study, it is also clear that no group-level traditions arose in the option-type chosen to solve the task. This is in contrast to a previous study [20] in which groups of meerkats could either use a flap, similar to the one in this study, to gain access to food in a box, or they could climb stairs to gain access to the food from above (henceforth, the "Flap/Stairs task"), and in which there was stronger evidence of group-level traditions arising through preferential adoption by observers of demonstrators' techniques. The SMFM presented in this paper provides a means by which we can interpret why traditions arise in some cases, but not in others: here we compare the present study (henceforth, the "Flap/Tube task") and the Flap/Stairs task. For the Flap/Tube task, we found the dominant effect was a transient local enhancement effect, which acted to attract observers to the specific option used by another individual. However, there was also evidence that this effect generalised to other nearby locations, including the option-type not used by the observed individual, especially in adults. We hypothesised that this effect may have been more option-type specific in the Flap/Stairs task.

We aimed to fit the same stochastic model of interaction with the task to the Flap/Stairs data (Eqn. 17), allowing us to estimate and compare the specificity of the estimated transient effects for each task. However, there were some differences for the Flap/Stairs task experiment and data collection that meant some modifications had to be made. First, only one box was presented, so we could not separate the effects of box-level local enhancement and stimulus enhancement from that of the general effect and specific local enhancement, so the model included only parameters for these latter two effects. This was accounted for in the calculations of specificity (see below). Secondly, it was not recorded whether each observer of a task manipulation saw the manipulator gain access to the box, so we assumed that any observation of a successful manipulation resulted in direct social learning. Since the main aim of the model was to compare the specificity of the transient social effects, this is unlikely to have biased the results. Thirdly, if an observer was seen to go on to manipulate the task without leaving the task area, the observation and manipulation were recorded as occurring within the same bout. Consequently, here we assumed that manipulation occurred directly after observation. This means any estimates of the half-life of the effect are likely to be an

underestimate, but given that we are interested only in the relative strength of option-specific and non-option-specific effects, this is not an important concern.

As a measure of specificity, we calculated from the model parameters the probability that a naïve observer chooses the same option-type as the individual it has observed, given it manipulates one of the options directly after observation, i.e. when the transient effects were strongest (as predicted by the transient effect sizes estimated by the SMFM, and ignoring the effects of direct social learning). This is simply: Rate of manipulating the same option-type/ Rate of manipulating any option-type. Since there are different baseline rates of manipulation for each option-type, we averaged over the option-types in each case. For instance, for adults in the Flap/Tube task, the rate of manipulating the same flap immediately after observing a flap manipulation is

$\theta_{SLE} + \theta_{BLE} + \theta_{SE} + \theta_{GE} + \exp(\mu_1)$,

and the rate of manipulating the other flap immediately after observing a flap manipulation is

$\theta_{SE} + \theta_{GE} + \exp(\mu_1)$.

Therefore, the total rate of flap manipulation, after observing a flap manipulation is:

$\theta_{SLE} + \theta_{BLE} + 2\theta_{SE} + 2\theta_{GE} + 2\exp(\mu_1)$,

and likewise the total rate of tube manipulation, after observing a tube manipulation is:

$\theta_{SLE} + \theta_{BLE} + 2\theta_{SE} + 2\theta_{GE} + 2\exp(\mu_2)$.

Averaging these gives us:

$\theta_{SLE} + \theta_{BLE} + 2\theta_{SE} + 2\theta_{GE} + \exp(\mu_1) + \exp(\mu_2)$

as the rate of manipulation of the same option-type. By similar logic, the rate of manipulating the other option-type is:

$\theta_{BLE} + 2\theta_{GE} + \exp(\mu_1) + \exp(\mu_2)$,

giving us a specificity of:

$$\frac{\theta_{SLE} + \theta_{BLE} + 2\theta_{SE} + 2\theta_{GE} + \exp(\mu_1) + \exp(\mu_2)}{\theta_{SLE} + 2\theta_{BLE} + 2\theta_{SE} + 4\theta_{GE} + 2\exp(\mu_1) + 2\exp(\mu_2)}. \qquad 23$$

The equivalent specificity for the Flap/Stairs tasks is:

$$\frac{\theta_{SLE} + \theta_{GE} + \frac{1}{2}\exp(\mu_1) + \frac{1}{2}\exp(\mu_2)}{\theta_{SLE} + 2\theta_{GE} + \exp(\mu_1) + \exp(\mu_2)}. \qquad 24$$

Equivalent expressions were used to calculate specificities for pups and juveniles/sub-adults.

When the option-type specific transient effects, such as specific local enhancement, are high relative to the total magnitude of all effects, we would expect specificity to be close to 1. This would indicate that a naïve observer will almost certainly chose the same option type as observed, if it interacts with a flap or a tube soon afterwards. When the option-type specific transient effects are small compared to the total magnitude of all effects, we would expect specificity to be close to 0.5. This would indicate that a naïve observer is no more likely to choose the same option-type as observed. In addition, even if the option-type specific effects are large compared to the total transient effect, specificity will be low if the baseline rates of interaction are relatively high, since this means social effects will be "drowned out" by observers' spontaneous interactions with the task.

We calculated specificities for each iteration of the MCMC sample (excluding the burn-in period), giving us an approximate posterior sample of the median specificity metric for each

age class in each case. We also subtracted the specificity yielded for each iteration for the Flap/Stairs task from the corresponding iteration for the Flap/Tube task, giving us a posterior sample for the difference in specificity. A summary of results is given in Table S8.

	Specificity for Flap/Stairs	Specificity for Flap/Tube	Difference in specificity Flap/Stairs – Flap/Tube
Adults	1.0<br>[0.98,1.0]	0.56<br>[0.28,0.86]	0.43<br>[0.13,0.72]<br>**<0.001**
Juveniles/Sub-adults	1.0<br>[0.99,1.0]	0.96<br>[0.88,1.0]	0.045<br>[-0.0029, 0.14]<br>**0.040**
Pups	1.0<br>[1.0,1.0]	0.79<br>[0.67,0.94]	0.20<br>[0.060, 0.33]<br>**0.002**

Table S8. The median and 95% HPD interval of the posterior sample for the specificity of the transient effect for the Flap/Tube and Flap/Stair tasks and the difference in specificity between the two tasks, for different age classes. Figures given in bold give the posterior probability that the specificity was less in the Flap/Stairs task for that age category.

The results suggest that the transient effect of observation was considerably more option-type specific for the Flap/Stairs task for pups and adults, than for the Flap/Tube task. Greater option-type specificity is therefore likely to account for the finding that group-level traditions were evident in the Flap/Stairs task but not in the Flap/Tube task. In the Flap/Tube task those adults and pups that are attracted to the alternative option type will rapidly erode any initial group bias towards either option-type. Further work is needed to investigate whether the specificity of transient social effects are generally important in determining the emergence of traditions, both in meerkats and other species, and also to investigate the factors (e.g. task design) that determine the specificity of transient social effects.

SUPPLEMENTAL REFERENCES

41. Gelman, A., et al., (2004) *Bayesian Data Analysis*. 2nd ed. CRC, Boca Raton.
42. Ntzoufras I (2009) *Bayesian Modeling Using WinBUGS*. John Wiley & Sons, New Jersey.
43. Jaynes E.T. (2003) *Probability Theory: The Logic of Science*. Cambridge University Press, Cambridge.
44. Johnson, D.H. (1998) The insignificance of statistical significance testing. *Journal of Wildlife Management* 63: 763-772.
45. Therneau, T. & Lumley T. *survival: Survival analysis, including penalised likelihood* (2009).
46. Fox, J. (2002) *Cox Proportional-Hazards Regression for Survival Data,* online appendix to *An R and S-PLUS Companion to Applied Regression*. Sage Publications: London.
