# Supplementary material for: Identification of Learning Mechanisms in a Wild Meerkat Population
Source: PLoS One. 2012 Aug 8;7(8):e42044. doi: 10.1371/journal.pone.0042044 (PMC3414518; doi:10.1371/journal.pone.0042044)
Supplement: Table S1 — Descriptive statistics of MCMC samples of the posterior distribution for parameters and contrasts in the final model. 95% credible intervals are highest posterior density (HPD). Where relevant the posterior probability is given that the parameter or contrast ≤0. These are not given in cases where parameters were constrained to be >0 or where the hypothesis = 0 is of no interest. Posterior probabilities are given to 3 d.p and in bold when <0.025. All other figures are given to 3 sig. figs. (PDF) [file pone.0042044.s005.pdf]

Effect	Mean	Median	SD	95% credible interval (HPD)	Posterior probability (≤0)
**Specific local enhancement:**					
Adults (θ_{SLE})	4.1E-4	3.4E-4	9.0E-4	[-0.0012, 0.0022]	0.342
Juveniles & Sub-adults ($\theta_{SLE,juvsub}$)	0.0050	0.0050	0.0012	[0.0028, 0.0074]	**<0.001**
Pups ($\theta_{SLE,pup}$)	0.0036	0.0035	0.0011	[0.0016, 0.0057]	**<0.001**
Contrast ($\theta_{SLE,juvsub}$ - θ_{SLE})	0.0046	0.0045	0.0014	[0.0019, 0.0074]	**<0.001**
Contrast ($\theta_{SLE,pup}$ - θ_{SLE})	0.0032	0.0031	0.0013	[7.1E-4, 0.0060]	**0.007**
Contrast ($\theta_{SLE,juvsub}$ - $\theta_{SLE,pup}$)	0.0014	0.0014	0.0014	[-0.0015, 0.0043]	0.154
**Box-level local enhancement:**					
Adults & Pups (θ_{BLE})	9.0E-4	8.5E-4	4.4E-4	[1.1E-4, 0.0018]	**0.001**
Juveniles & Sub-adults ($\theta_{BLE,juvsub}$)	2.1E-4	1.6E-4	1.8E-4	[-2.0E-7, 5.6E-4]	**0.001**
Contrast (θ_{BLE} - $\theta_{BLE,juvsub}$)	7.0E-4	6.4E-4	4.2E-4	[-2.2E-5, 0.0016]	**0.018**
**General effect:**					
Adults & pups (θ_{GE})	1.7E-4	1.4E-4	4.7E-4	[0, 4.5E-4]	
Juveniles & Sub-adults ($\theta_{GE,juvsub}$)	3.6E-5	2.2E-5	4.3E-5	[0, 6.3E-5]	
Contrast (θ_{GE} - $\theta_{GE,juvsub}$)	1.4E-4	1.1E-4	1.3E-4	[-3.7E-6, 4.0E-4]	**0.017**
**Half-life of transient effect (secs) ($\log(2)/\beta$)**	20	20	4.5	[12, 29]	
**Direct social learning rate:**					
Option-specific (s)	0.0035	0.0035	6.6E-4	[0.0017, 0.0055]	
Cross-option (s_{cross})	0.0013	0.0012	9.9E-4	[1.0E-4, 0.0026]	
Contrast ($s - s_{cross}$)	0.0022	0.0022	1.2E-3	[-1.1E-4, 0.0045]	0.027
**Asocial learning rate**					
Subordinates (α)	0.051	0.051	5.7E-3	[0.040, 0.063]	
Dominants (α_{dom})	4.2E-4	5.5E-12	3.8E-3	[0, 9.9E-4]	
Contrast (α - α_{dom})	0.036	0.035	0.013	[0.013,0.060]	**0.002**
**Effect of learning (ω):**	0.0049	0.0049	2.1E-4	[0.0045, 0.0053]	
**Baseline rate effects (on scale of linear predictor):**					
Flap μ_1	-13	-13	0.52	[-14, -12]	
Difference between flap and tube ($\mu_2 - \mu_1$)	0.81	0.81	0.23	[0.37, 1.3]	**<0.001**
Male effect (κ_{male})	1.09	1.08	0.47	[0.18, 2.0]	**0.009**

Table S1: Descriptive statistics of MCMC samples of the posterior distribution for parameters and contrasts in the final model. 95% credible intervals are highest posterior density (HPD). Where relevant the posterior probability is given that the parameter or contrast ≤0. These are not given in cases where parameters were constrained to be >0 or where the hypothesis =0 is of no interest. Posterior probabilities are given to 3 d.p and in bold when <0.025. All other figures are given to 3 sig. figs
