# Supplementary material for: Identification of Learning Mechanisms in a Wild Meerkat Population
Source: PLoS One. 2012 Aug 8;7(8):e42044. doi: 10.1371/journal.pone.0042044 (PMC3414518; doi:10.1371/journal.pone.0042044)
Supplement: Table S2 — Relative support for different models of the effect of direct social learning on rate of solving. (DOC) [file pone.0042044.s006.doc]

|  | Total Akaike weight (%) | |
| --- | --- | --- |
| No social learning | 18.2 | |
| Conditions for social learning: | Option-specific | Option-general |
| All observations | 8.6 | 8.1 |
| Observations of reward | 7.6 | 9.9 |
| Observations of box entry | 7.2 | 8.4 |
| Observations of both box entry and reward | 7.2 | 24.5 |

Table S2. Relative support for different models of the effect of direct social learning on rate of solving.
