# Supplementary material for: Identification of Learning Mechanisms in a Wild Meerkat Population
Source: PLoS One. 2012 Aug 8;7(8):e42044. doi: 10.1371/journal.pone.0042044 (PMC3414518; doi:10.1371/journal.pone.0042044)
Supplement: Table S3 — Relative support for different models of the effect of direct social learning on rate of task abandonment. (DOC) [file pone.0042044.s007.doc]

|  | Total Akaike weight (%) | |
| --- | --- | --- |
| No social learning | 0.8 | |
| Conditions for social learning: | Option-specific | Option-general |
| All observations | 0.5 | 1.2 |
| Observations of reward | 0.4 | 3.9 |
| Observations of box entry | 0.4 | 0.6 |
| Observations of both box entry and reward | 5.9 | 86.3 |

Table S3. Relative support for different models of the effect of direct social learning on rate of task abandonment.
